# Supplementary material for: Meroterpenoids and Steroids from the Marine-Derived Fungus Trametes sp. ZYX-Z-16
Source: Molecules. 2022 Dec 11;27(24):8782. doi: 10.3390/molecules27248782 (PMC9784055; doi:10.3390/molecules27248782)
Supplement: Supplementary file 1 [file molecules-27-08782-s001.zip › molecules-2038189-supplementary.pdf]

Supporting Information

Meroterpenoids and steroids from the marine-derived fungus *Trametes* sp. ZYX-Z-16

Zi Ming Ren[1,†], Li Yang[2,†], Qing Yun Ma[2], Qing Yi Xie[2], Hao Fu Dai[2], Kun Lai Sun[1,⊥] and You Xing Zhao[2],*

1 Zhejiang Provincial Engineering Technology Research Center of Marine Biomedical Products, School of Food and Pharmacy, Zhejiang Ocean University, Zhoushan 316022, China

2 Haikou Key Laboratory for Research and Utilization of Tropical Natural Products, Institute of Tropical Bioscience and Biotechnology, Chinese Academy of Tropical Agricultural Sciences & Hainan Key Laboratory for Protection and Utilization of Tropical Bioresources, Hainan Academy of Tropical Agricultural Resource, Haikou 571101, China

† These authors contributed equally to this work.

* Correspondence author.

sunqinlai@126.com (K.L.S); zhaoyouxing@itbb.org.cn (Y.X.Z.)

Content

ITS1 gene sequences of *Trametes* sp. ZYX-Z-16..4

Figure S1. Original tree of *Trametes* sp. ZYX-Z-16..4

Figure S2. ^{1}H-NMR spectrum of compound **1** (500 MHz, CD_3OD)............................5

Figure S3. ^{13}C-NMR and DEPT spectra of compound **1** (125 MHz, CD_3OD) 5

Figure S4. HSQC spectrum of compound **1** (125 MHz, CD_3OD)...............................6

Figure S5. HMBC spectrum of compound **1** (125 MHz, CD_3OD)...............................6

Figure S6. ^{1}H-^{1}H COSY spectrum of compound **1** (125MHz, CD_3OD) 7

Figure S7. ROESY spectrum of compound **1** (125 MHz, CD_3OD).............................7

Figure S8. HRESIMS of compound **1** (MeOH)...8

Figure S9. IR spectrum of the compound **1** 8

Figure S10. ^{1}H-NMR spectrum of compound **2** (500 MHz, $CDCl_3$)............................9

Figure S11. ^{13}C-NMR and DEPT spectra of compound **2** (125 MHz, $CDCl_3$).............9

Figure S12. HSQC spectrum of compound **2** (125 MHz, $CDCl_3$).............................10

Figure S13. HMBC spectrum of compound **2** (125 MHz, $CDCl_3$)............................10

Figure S14. ^{1}H-^{1}H COSY spectrum of compound **2** (125MHz, $CDCl_3$) 11

Figure S15. ROESY spectrum of compound **2** (125 MHz, $CDCl_3$)...........................11

Figure S16. HRESIMS of compound **2** (MeOH)...12

Figure S17. IR spectrum of the compound **2** 12

Table S1. Experimental and calculated ^{13}C NMR chemical shifts of **1.** 13

Table S2. Experimental and calculated ^{1}H NMR chemical shifts of **1.**.......................13

Figure S18. DP4+ analyses of calculated and experimental NMR chemical shifts of 20*R*-**1** and 20*S*-**1.**...15

Table S3. Conformational analysis of the optimized isomers of **1** in methanol..........15

Figure S19. Optimized conformers of compound **1**...16

Table S4. Optimized cartesian coordinates of conformers of (5*R*,7*S*,8*S*,9*S*,*10S*,1′*R*,5′*S*,6′*S*)-**1** at B3LYP/6-311G(d) level in methanol**.**..................16

Table S5. Optimized cartesian coordinates of conformers of (5*R*,7*S*,8*S*,9*S*,10*S*,1′*S*,5′*R*,6′*R*)-**1** at B3LYP/6-311G(d) level in methanol**.** 18

Table S6. Conformational analysis of the optimized isomers of **2** in chloroform.......20

Figure S20. Optimized conformers of compound **2**..20

Table S7. Optimized cartesian coordinates of conformers of **2** at B3LYP/6-311G(d) level in chloroform...20

Table S8. α-Glucosidase and acetylcholinesterase inhibitory activities of all compounds (IC_{50}, μM) 24

Table S9. Antibacterial activities of all compounds MIC (μg/mL) 24

Table S10. Antifungal activities of all compounds MIC (μg/mL)..............................25

ITS1 gene sequences of *Trametes* sp. ZYX-Z-16

GGCCTACGAGTGCGGGACTCGCGGTCACCTCCCACCCGTGTCTCTCTCTAC
CTGTTGCTTTGGCGGGCCCACCGGGGCCACCCGGTCGCCGGGGGACGTCG
TCCCCGGGCCCGCGCCCGCCGAAGCGCTCTGTGAACCCTGATGAAGATGG
GCTGTCTGAGTCGAATGAAAATTGTCAAAACTTTCAACAATGGATCTCTTG
GTTCCGGCATCGATGAAGAACGCAGCGAAATGCGATAAGTAATGTGAATTG
CAGAATTCCGTGAATCATCGAATCTTTGAACGCACATTGCGCCCCCTGGCAT
TCCGGGGGGCATGCCTGTCCGAGCGTCATTTCTGCCCTCAAGCCCGGCTTG
TGTGTTGGGCGTGGTCCCCCCGGGGACCTGCCCGAAAGGCAGCGGCGACG
TCCGTCTGGTCCTCGAGCGTATGGGGCTCTGTCACTCGCTCGGGACGGATC
GGCGGAGGTTGGTCACCACCACAGTTTTACCACGGTTGACCTCGGATCAG
GTAGGAGTTACCCGCTGAACTTAAGCATATCAAAG

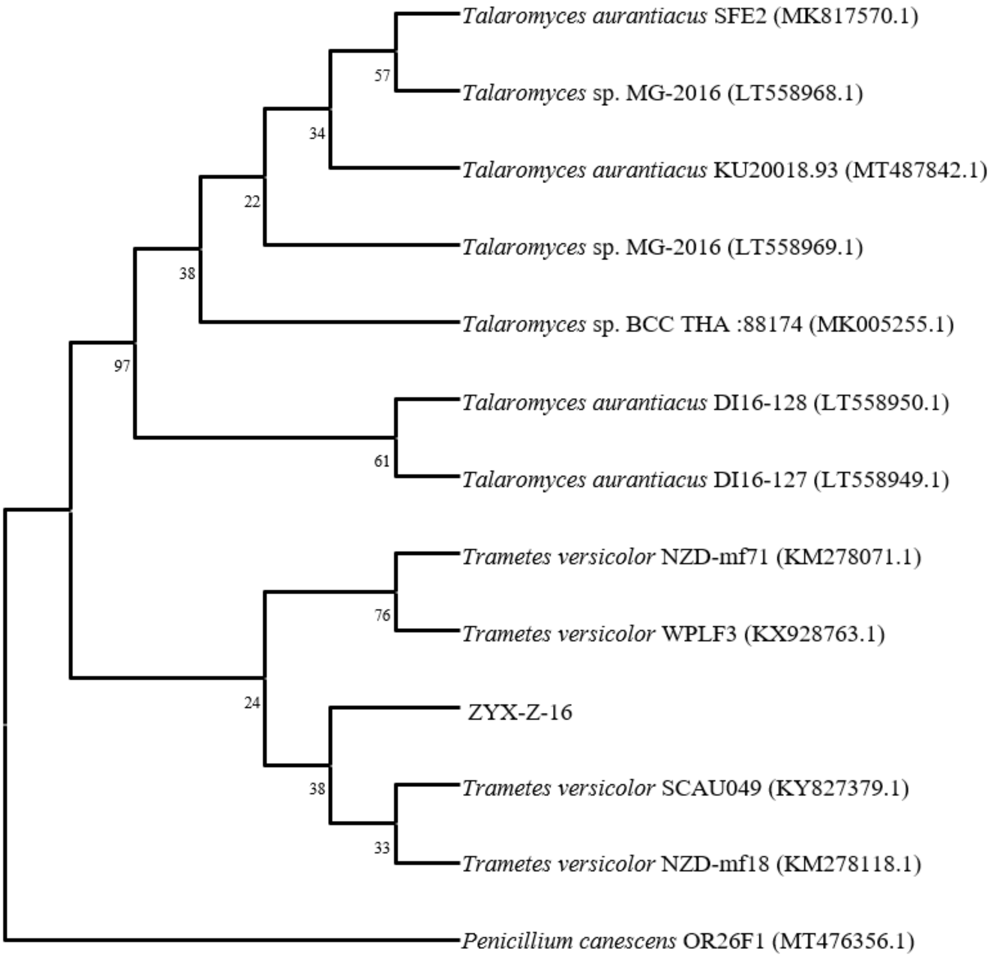

Figure S1. Original tree of *Trametes* sp. ZYX-Z-16

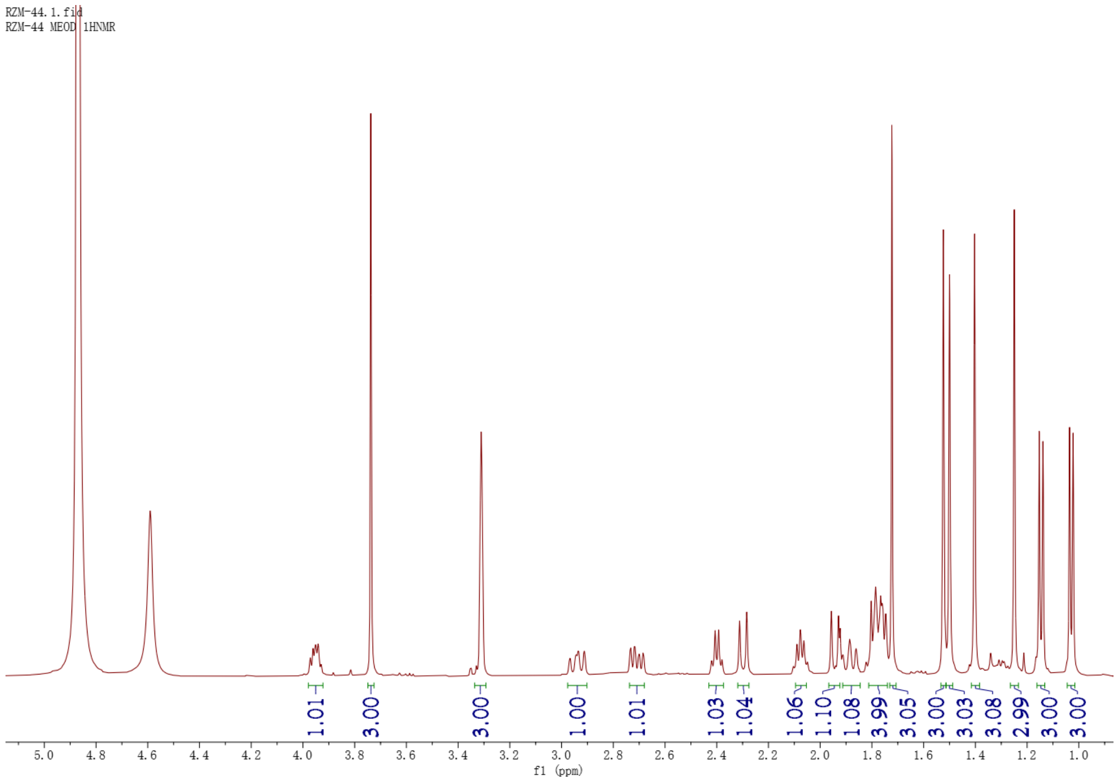

Figure S2.^{1}H-NMR spectrum of compound **1** (500 MHz, CD_3OD)

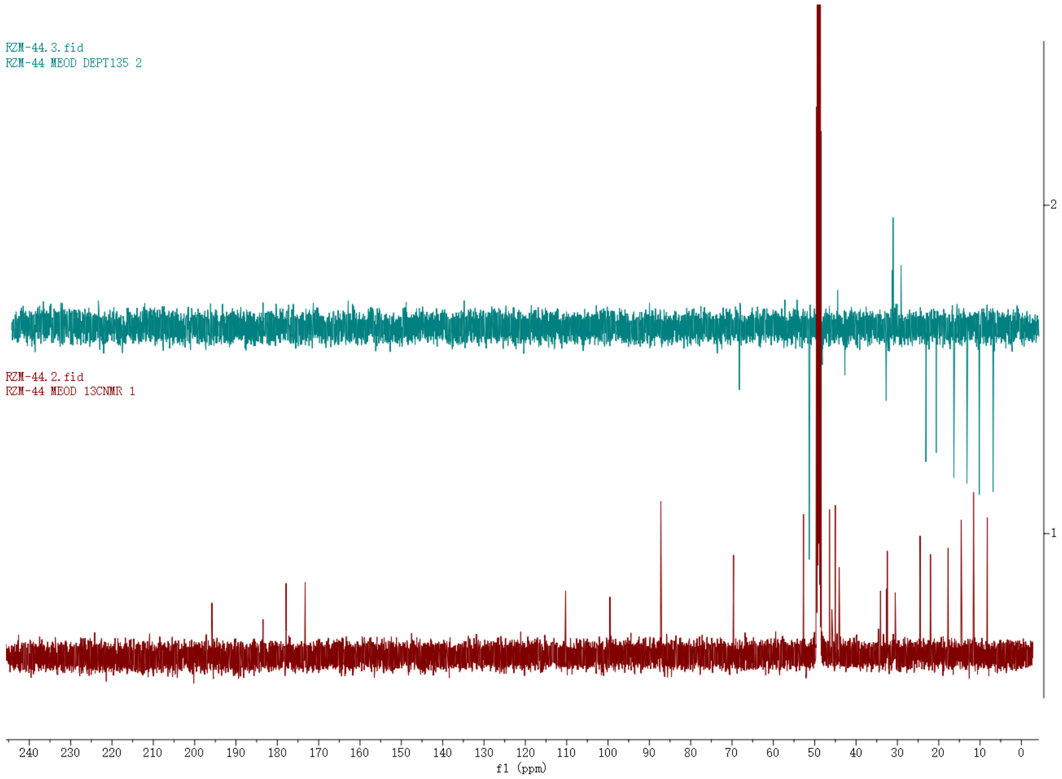

Figure S3.^{13}C-NMR and DEPT spectra of compound **1** (125 MHz, CD_3OD)

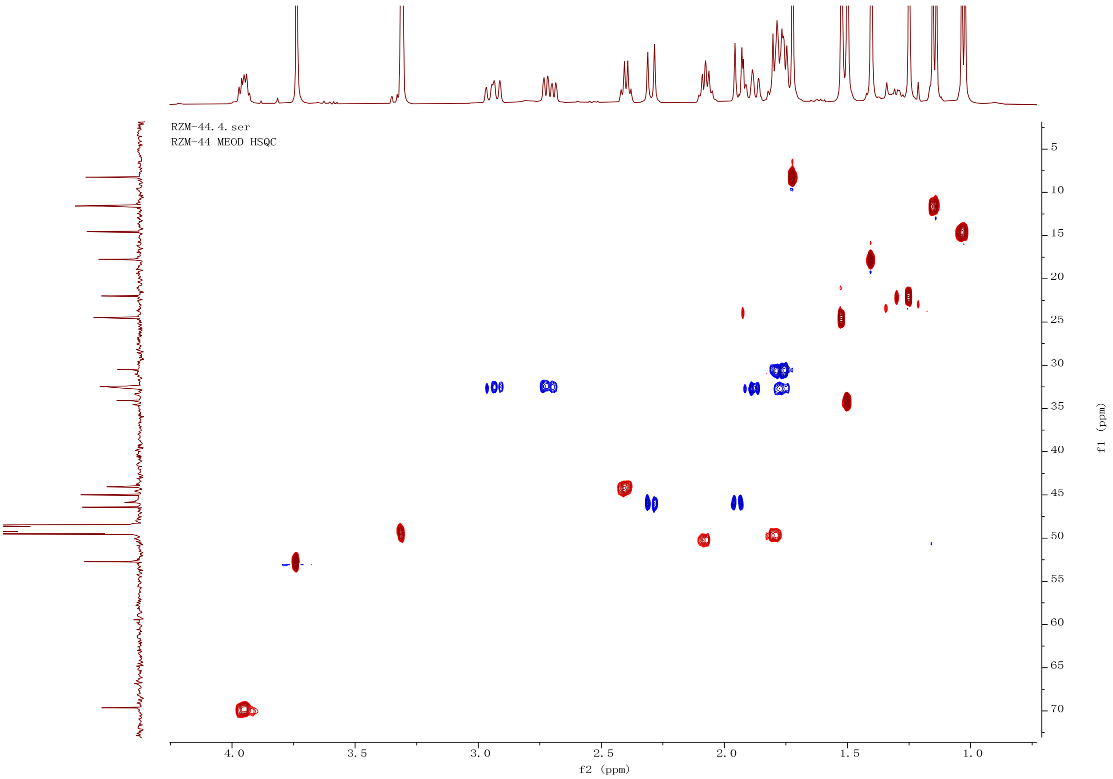

Figure S4. HSQC spectrum of compound **1** (125 MHz, CD_3OD)

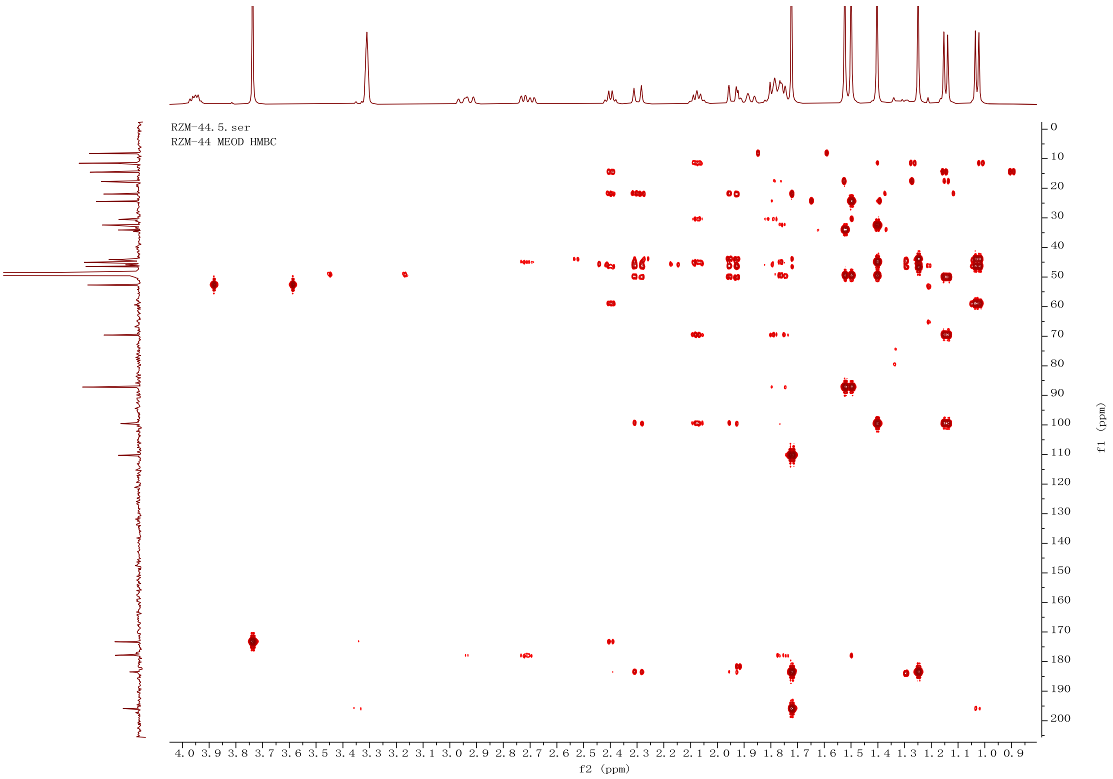

Figure S5. HMBC spectrum of compound **1** (125 MHz, CD_3OD)

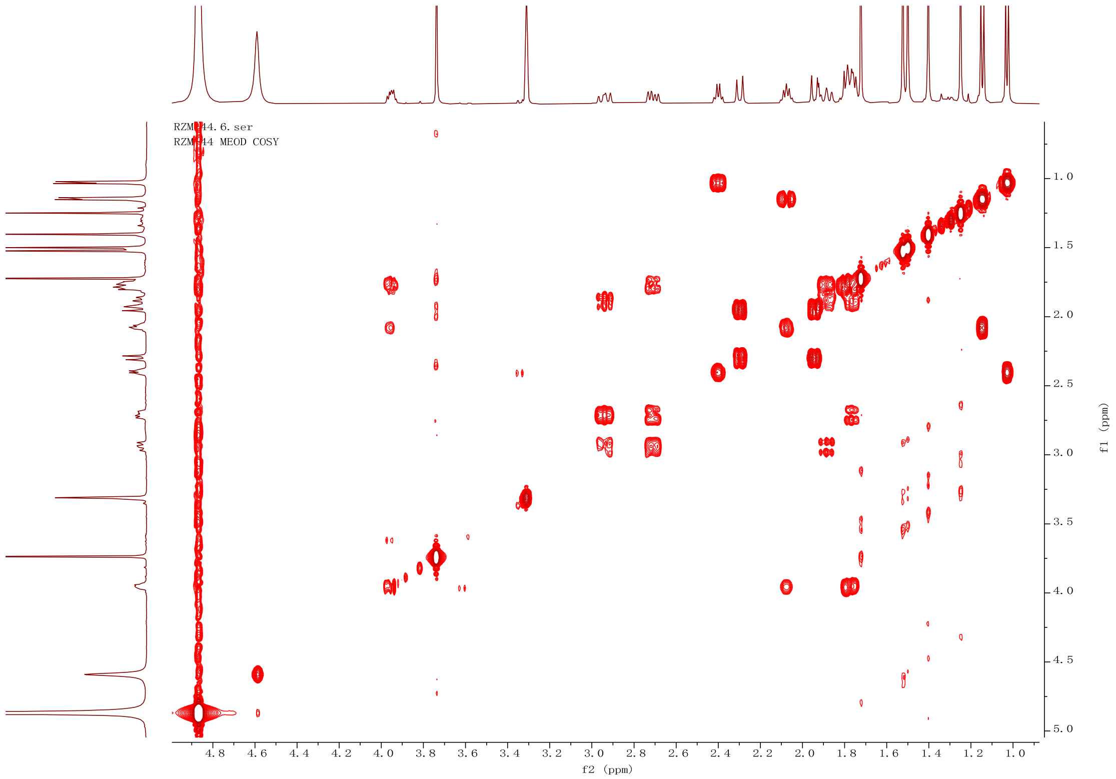

Figure S6. ^{1}H-^{1}H COSY spectrum of compound **1** (125MHz, CD_3OD)

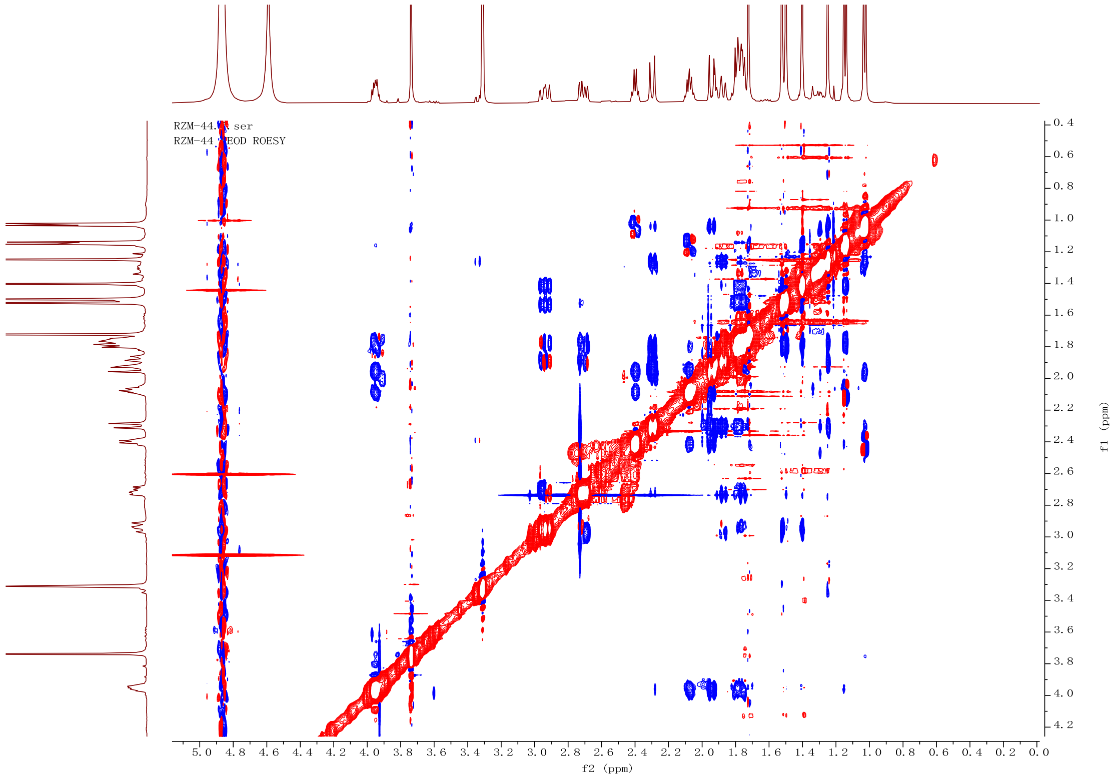

Figure S7. ROESY spectrum of compound **1** (125 MHz, CD_3OD)

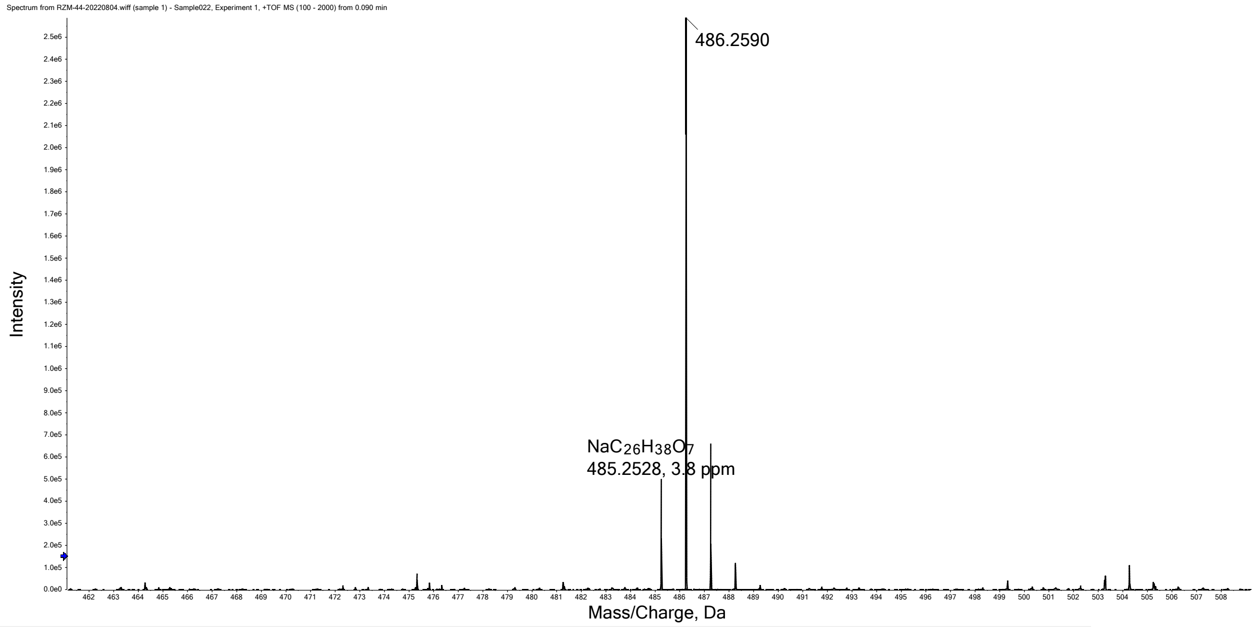

Hit	Formula	m/z	RDB	ppm	MS Rank	MSMS ppm	MSMS Rank	Found
1	C26H38O7	485.2510	8.0	3.8	1			NA/NA

Figure S8. HRESIMS of compound **1** (MeOH)

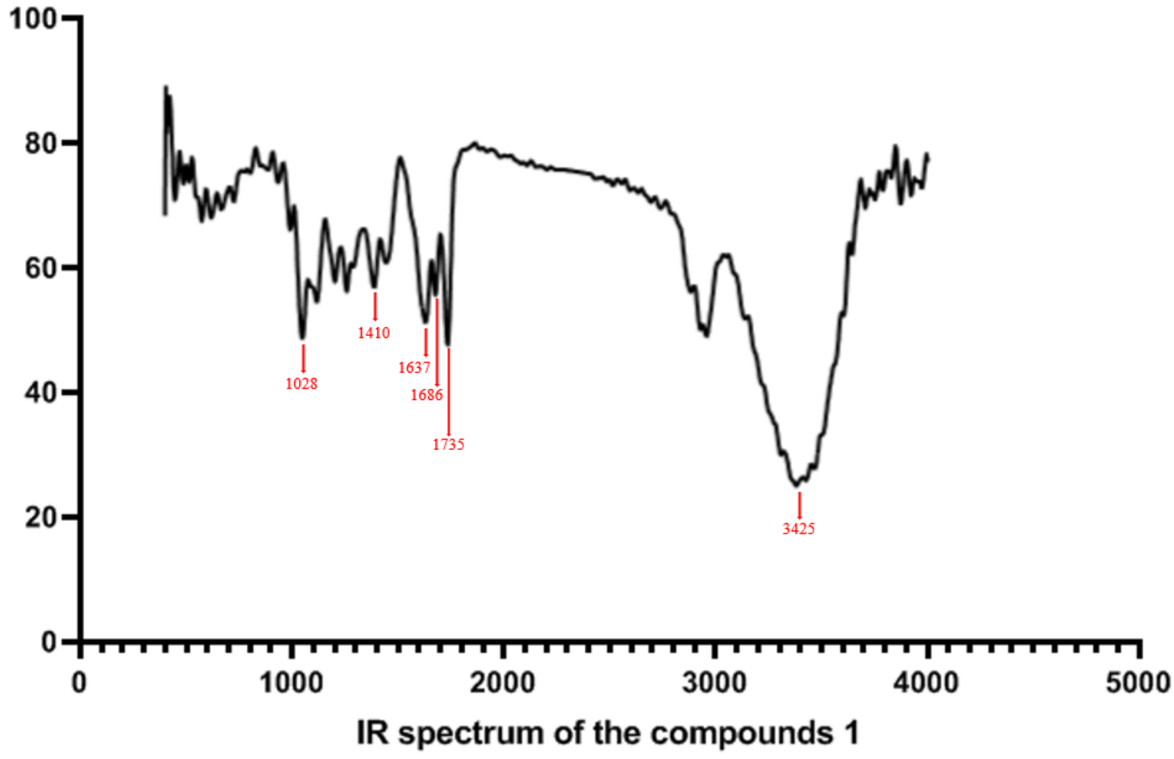

Figure S9. IR spectrum of the compound **1**

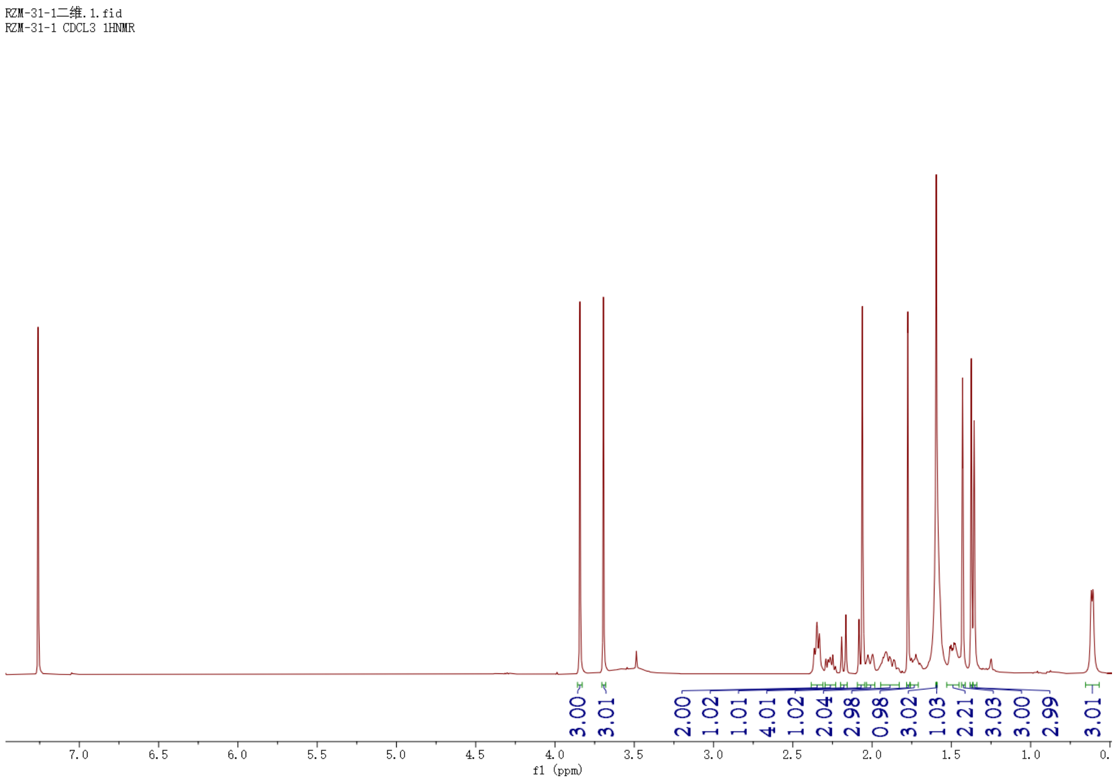

Figure S10.^{1}H-NMR spectrum of compound **2** (500 MHz, CDCl$_3$)

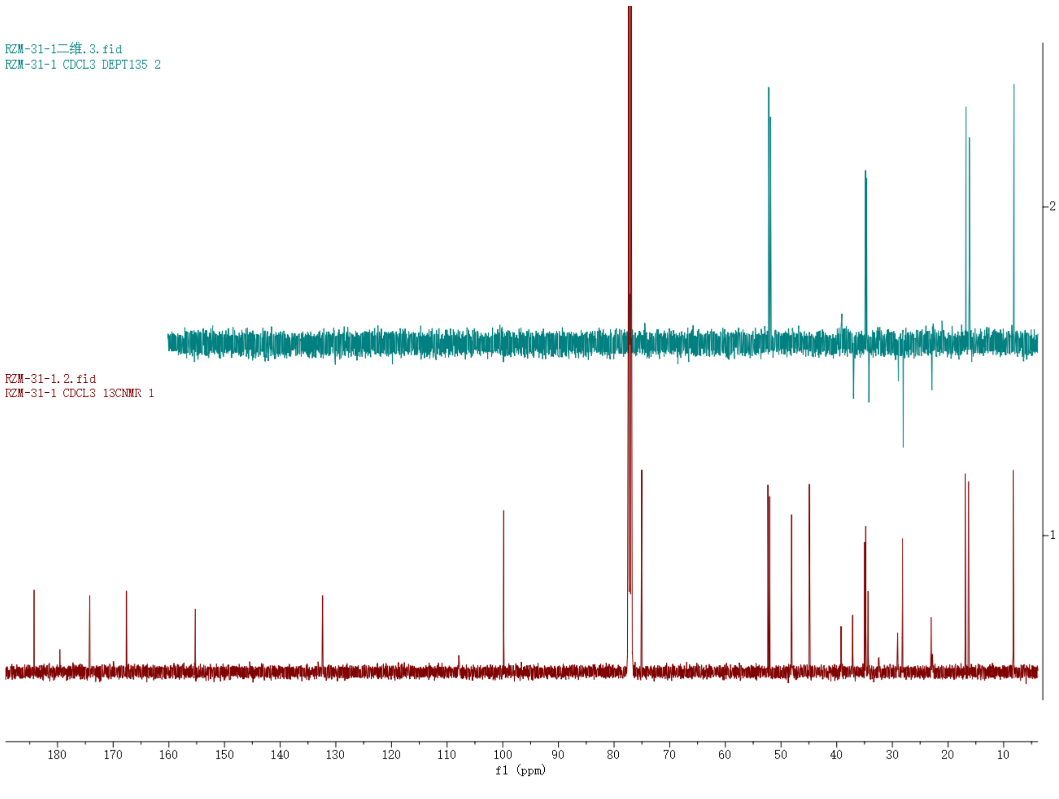

Figure S11.^{13}C-NMR and DEPT spectra of compound **2** (125 MHz, CDCl$_3$)

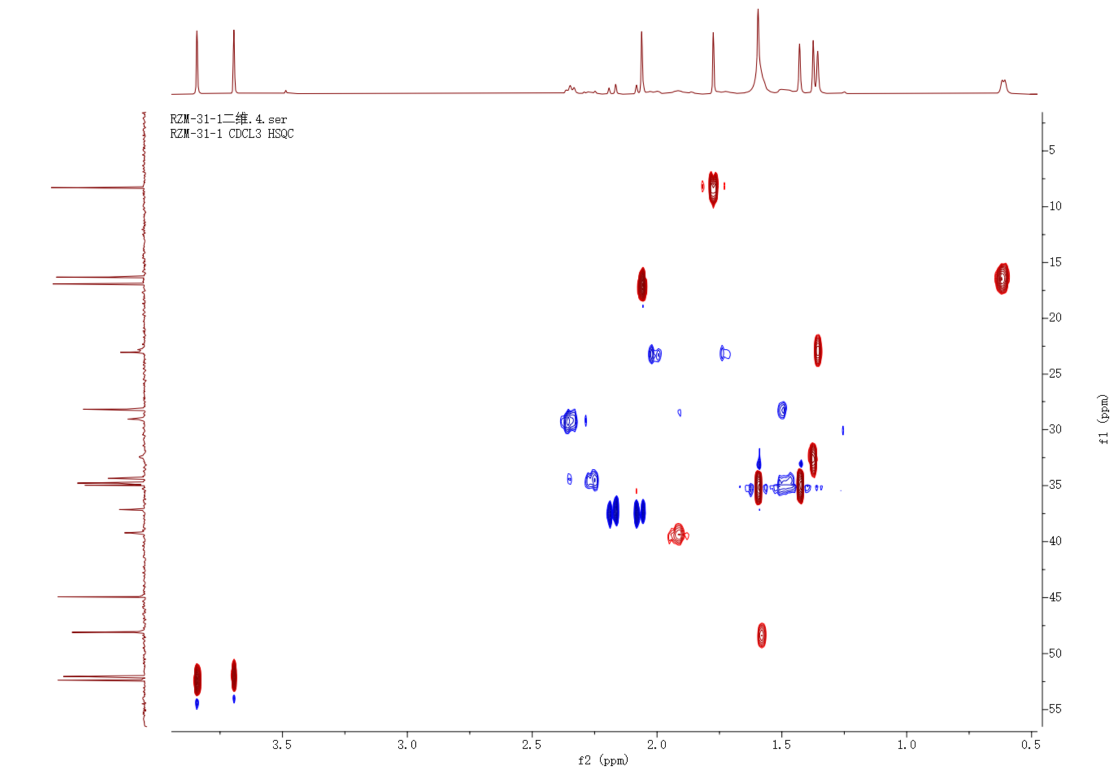

Figure S12. HSQC spectrum of compound **2** (125 MHz, $CDCl_3$)

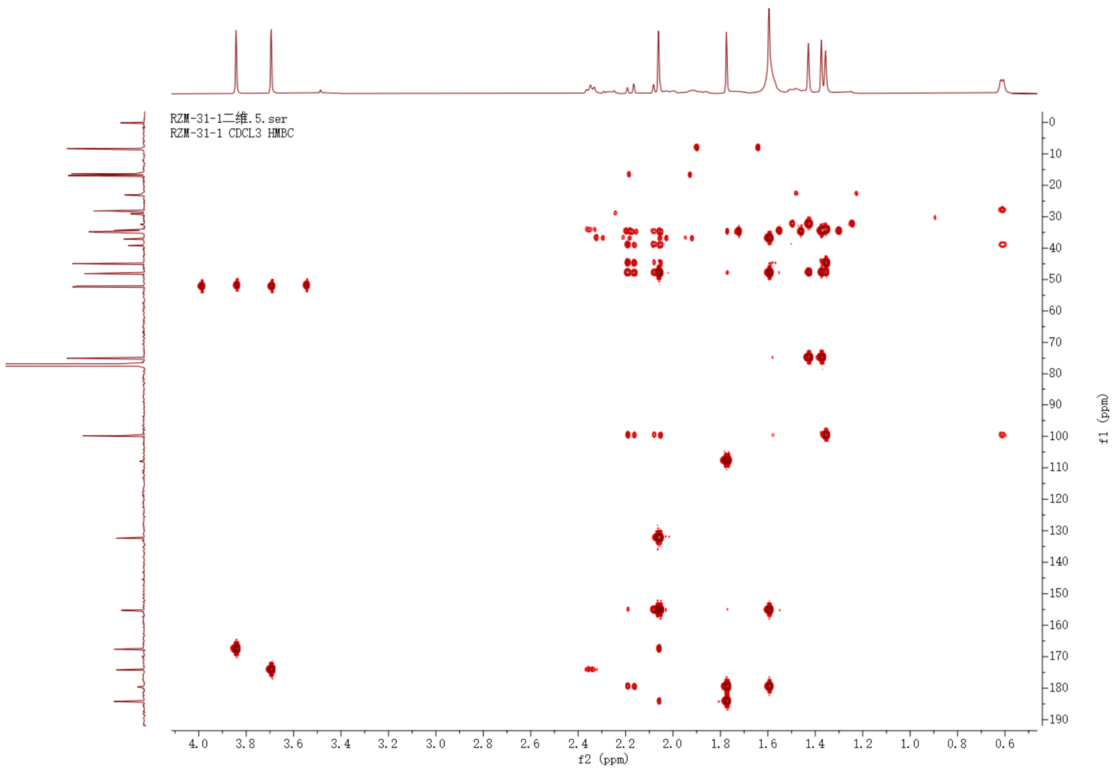

Figure S13. HMBC spectrum of compound **2** (125 MHz, $CDCl_3$)

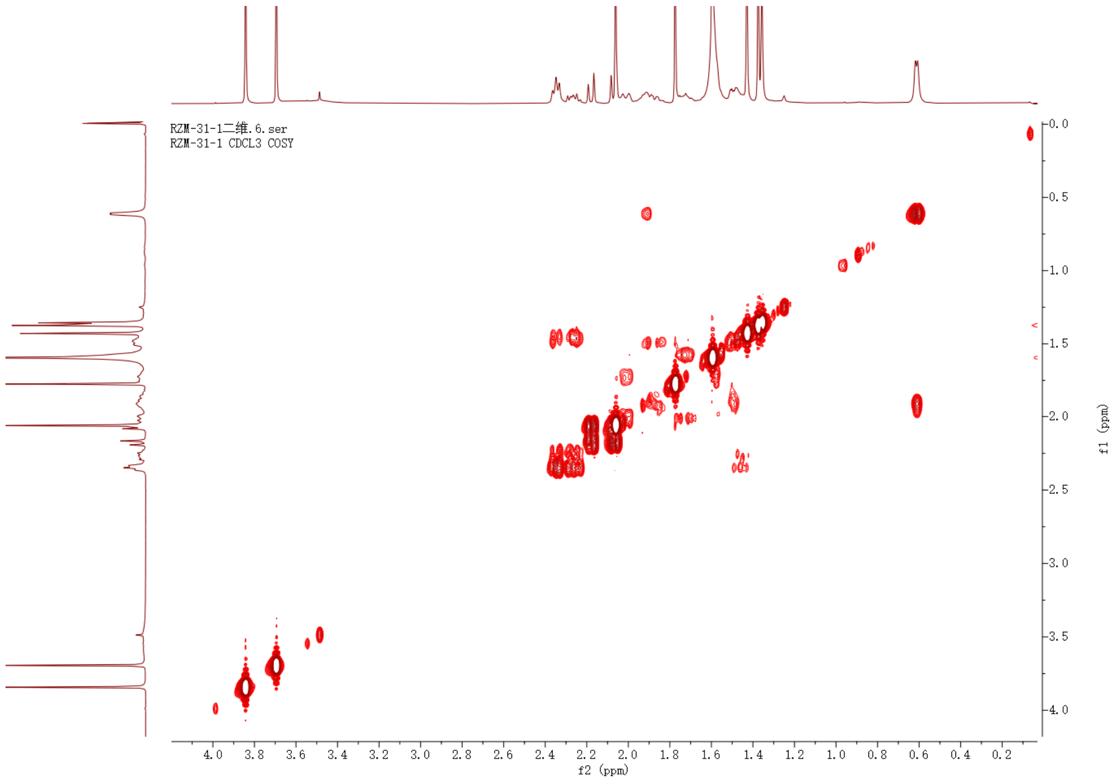

Figure S14. ^{1}H-^{1}H COSY spectrum of compound **2** (125MHz, $CDCl_3$)

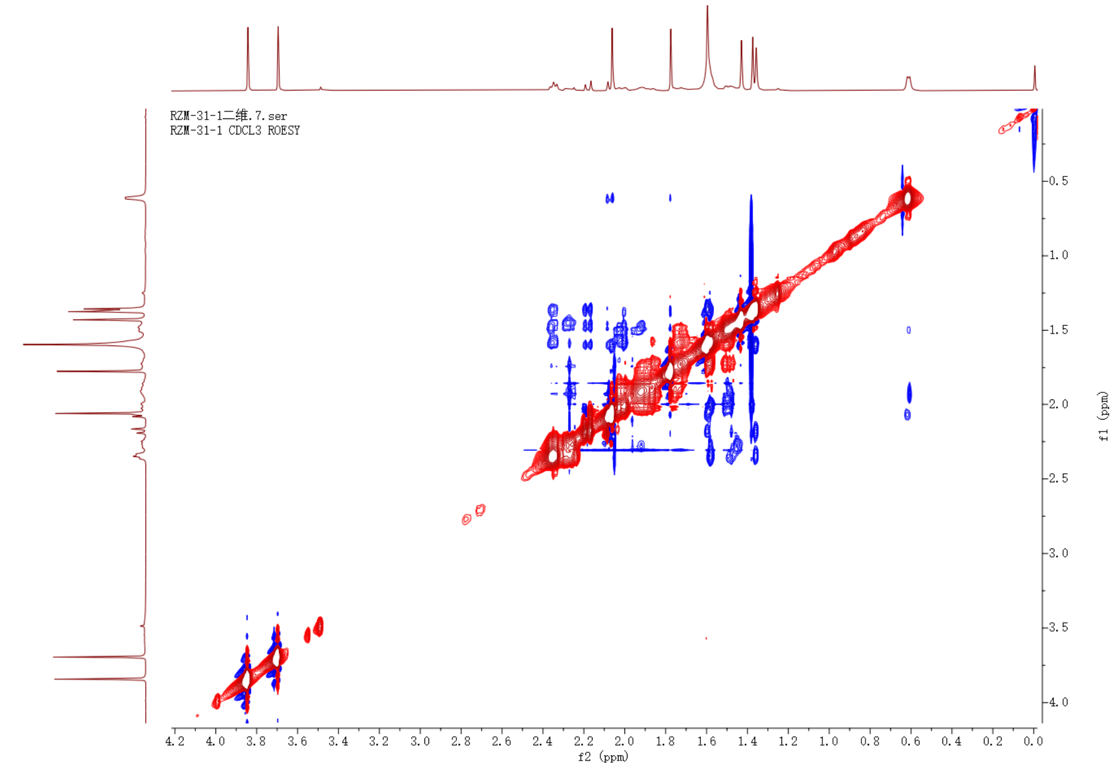

Figure S15. ROESY spectrum of compound **2** (125 MHz, $CDCl_3$)

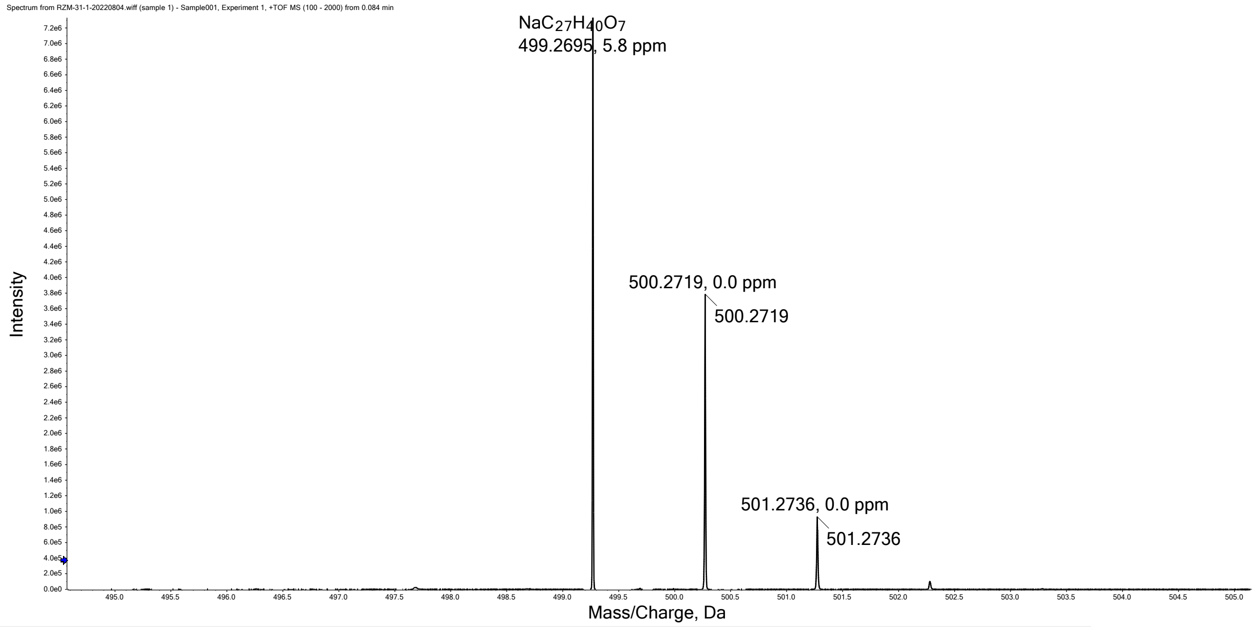

Hit	Formula	m/z	RDB	ppm	MS Rank	MSMS ppm	MSMS Rank	Found
1	C27H40O7	499.2666	8.0	5.8	1			NA/NA

Figure S16. HRESIMS of compound **2** (MeOH)

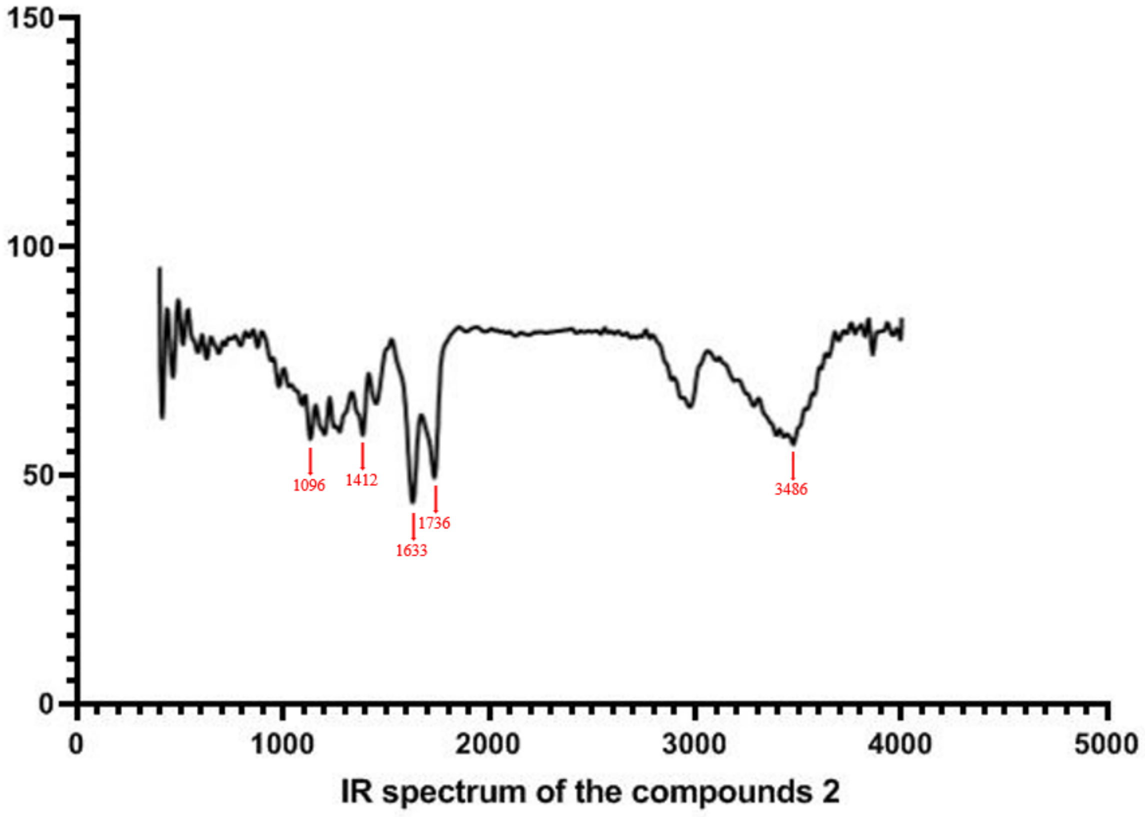

Figure S17. IR spectrum of the compound **2**

Table S1. Experimental and calculated ^{13}C NMR chemical shifts of **1.**

No.	Experimental (δ_C, ppm)	Calculated (δ_C, ppm)		Corrected (δ_C, ppm)	
		(5*R*,7*S*,8*S*,9*S*,*10S*, 1′*R*,5′*S*,6′*S*)-**1**	(5*R*,7*S*,8*S*,9*S*,10*S*, 1′*S*,5′*R*,6′*R*)-**1**	(5*R*,7*S*,8*S*,9*S*,10*S*, 1′*R*,5′*S*,6′*S*)-**1**	(5*R*,7*S*,8S,9S,10*S*, 1′*S*,5′*R*,6′*R*)-**1**
1	32.6	33.6	32.7	31.8	30.9
2	32.4	34.6	34.8	32.9	33.0
3	177.9	173.5	173.2	175.9	175.9
4	87.2	87.9	87.7	87.8	87.6
5	49.3	50.5	49.7	49.2	48.4
6	30.5	31.4	32.3	29.6	30.4
7	69.6	71.5	72.6	70.9	72.0
8	50.1	53.2	50.9	52.0	49.7
9	99.5	100.4	99.8	100.6	100.1
10	45	47.7	51.0	46.4	49.7
11	45.9	46.7	46.8	45.4	45.4
12	11.6	13.4	13.0	11.1	10.5
13	17.7	18.7	19.7	16.5	17.4
14	34.1	34.8	35.1	33.1	33.3
15	24.5	24.3	24.5	22.2	22.3
1'	46.4	49.4	50.5	48.1	49.2
2'	183.4	181.2	181.9	183.9	184.8
3'	110.2	109.9	107.5	110.5	108.1
4'	195.8	191.8	191.9	194.8	195.2
5'	59.4	60.6	60.2	59.7	59.2
6'	44.1	46.6	44.4	45.2	42.9
7'	22	22.5	23.9	20.5	21.8
8'	8.2	12.0	11.9	9.6	9.4
9'	173.3	171.3	171.5	173.7	174.1
10'	14.6	16.0	16.2	13.7	13.8
11'	52.7	53.9	54.2	52.8	53.0
**R^2**		**0.9996**	**0.9992**		
**MAE**		**1.75**	**1.89**		
**CMAE**				**0.97**	**1.17**

Table S2. Experimental and calculated ^{1}H NMR chemical shifts of **1.**

No.	Experimental (δ_H, ppm)	Calculated (δ_H, ppm)		Corrected (δ_H, ppm)	
		(5*R*,7*S*,8*S*,9*S*,*10S*, 1′*R*,5′*S*,6′*S*)-**1**	(5*R*,7*S*,8*S*,9*S*,10*S*, 1′*S*,5′*R*,6′*R*)-**1**	(5*R*,7*S*,8*S*,9*S*,10*S*, 1′*R*,5′*S*,6′*S*)-**1**	(5*R*,7*S*,8S,9S,10*S*, 1′*S*,5′*R*,6′*R*)-**1**
1a	1.87	1.67	1.38	1.75	1.44

1b	1.77	1.60	1.53	1.68	1.58
2a	2.94	2.65	2.94	2.71	2.94
2b	2.71	2.52	2.44	2.58	2.46
5	1.8	1.76	1.65	1.83	1.70
6	1.77	1.79	1.68	1.86	1.73
7	3.95	4.07	4.00	4.10	3.96
8	2.07	2.00	2.53	2.07	2.55
11a	2.3	1.97	2.26	2.04	2.28
11b	1.94	1.85	1.78	1.93	1.83
12	1.14	1.05	1.24	1.14	1.30
13	1.4	1.33	1.38	1.41	1.44
14	1.5	1.41	1.48	1.49	1.53
15	1.52	1.46	1.44	1.54	1.49
5'	3.34	3.22	3.35	3.27	3.34
6'	2.4	2.56	2.53	2.61	2.55
7'	1.25	1.19	1.28	1.28	1.34
8'	1.72	1.80	1.76	1.87	1.81
10'	1.03	0.90	0.95	0.99	1.03
11'	3.74	3.77	3.88	3.81	3.85
**R^2**		**0.9798**	**0.9561**		
**MAE**		**0.11**	**0.13**		
**CMAE**				**0.09**	**0.12**

Functional		Solvent?		Basis Set		Type of Data	
mPW1PW91		PCM		6-31+G(d,p)		Unscaled Shifts	
		DP4+	100.00%	0.00%	-	-	-
Nuclei	sp2?	xperiments	Isomer 1	Isomer 2	Isomer 3	Isomer 4	Isomer 5
C		32.4	33.6	32.7			
C		30.5	34.6	34.8			
C	x	177.9	173.5	173.2			
C		87.2	87.9	87.7			
C		49.3	50.5	49.7			
C		32.6	31.4	32.3			
C		69.6	71.5	72.6			
C		50.1	53.2	50.9			
C		99.5	100.4	99.8			
C		45	47.7	51.0			
C		45.9	46.7	46.8			
C		11.6	13.44	13.01			
C		17.7	18.68	19.68			
C		34.1	34.81	35.10			
C		24.5	24.26	24.46			
C		46.4	49.39	50.47			
C	x	183.4	181.24	181.85			
C	x	110.2	109.91	107.53			
C	x	195.8	191.82	191.90			
C		59.4	60.58	60.21			
C		44.1	46.57	44.40			
C		22	22.53	23.94			
C		8.2	11.97	11.92			
C	x	173.3	171.35	171.52			
C		14.6	15.96	16.17			
C		52.7	53.888605	54.187539			
H		1.77	1.6390211	1.4558492			
H		2.94	2.6486251	2.9381852			
H		2.71	2.5196001	2.441481			
H		1.77	1.7550969	1.65251			
H		1.87	1.8451717	1.6728422			
H		1.78	1.7346034	1.6931898			
H		3.95	4.0708507	3.995408			
H		2.07	2.0001416	2.5298652			
H		2.3	1.9656205	2.2574624			
H		1.94	1.8518999	1.7843574			
H		1.14	1.05053817	1.23775373			
H		1.4	1.3258759	1.3840432			
H		1.5	1.40914513	1.47910367			
H		1.52	1.45950803	1.43834627			
H		3.34	3.2230952	3.3467094			
H		2.4	2.5552764	2.530666			
H		1.25	1.18921947	1.28290947			
H		1.72	1.79903093	1.76023087			
H		1.03	0.89979063	0.9529862			
H		3.74	3.7705932	3.88095287			

Functional	Solvent?		Basis Set		Type of Data	
mPW1PW91	PCM		6-31+G(d,p)		Unscaled Shifts	
	Isomer 1	Isomer 2	Isomer 3	Isomer 4	Isomer 5	Isomer 6
sDP4+ (H data)	98.69%	1.31%	-	-	-	-
sDP4+ (C data)	98.61%	1.39%	-	-	-	-
sDP4+ (all data)	99.98%	0.02%	-	-	-	-
uDP4+ (H data)	91.98%	8.02%	-	-	-	-
uDP4+ (C data)	99.20%	0.80%	-	-	-	-
uDP4+ (all data)	99.93%	0.07%	-	-	-	-
DP4+ (H data)	99.88%	0.12%	-	-	-	-
DP4+ (C data)	99.99%	0.01%	-	-	-	-
DP4+ (all data)	100.00%	0.00%	-	-	-	-

Figure S18. DP4+ analyses of calculated and experimental NMR chemical shifts of 20*R*-**1** and 20*S*-**1.**

Table S3. Conformational analysis of the optimized isomers of **1** in methanol.

Conformations	*G* (hartree)	*ΔG* (kcal/mol)	Boltzmann distributions (%)
(5*R*,7*S*,8*S*,9*S*,*10S*,1′*R*,5′*S*,6′*S*)-**1-1**	-1540.278906	0	47.9
(5*R*,7*S*,8*S*,9*S*,*10S*,1′*R*,5′*S*,6′*S*)-**1-2**	-1540.278754	0.095695	40.7
(5*R*,7*S*,8*S*,9*S*,*10S*,1′*R*,5′*S*,6′*S*)-**1-3**	-1540.277548	0.852103	11.4
(5*R*,7*S*,8*S*,9*S*,10*S*,1′*S*,5′*R*,6′*R*)-**1-1**	-1540.280724	0	34.1

(5*R*,7*S*,8*S*,9*S*,10*S*,1′*S*,5′*R*,6′*R*)-**1-2**	-1540.280635	0.056413149	31.1
(5*R*,7*S*,8*S*,9*S*,10*S*,1′*S*,5′*R*,6′*R*)-**1-3**	-1540.2804	0.203752497	24.2
(5*R*,7*S*,8*S*,9*S*,10*S*,1′*S*,5′*R*,6′*R*)-**1-4**	-1540.279625	0.690072747	10.6

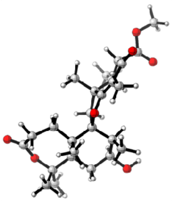
(5*R*,7*S*,8*S*,9*S*,*10S*, 1′*R*,5′*S*,6′*S*)-1-1

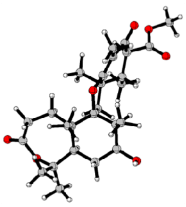
(5*R*,7*S*,8*S*,9*S*,*10S*, 1′*R*,5′*S*,6′*S*)-1-2

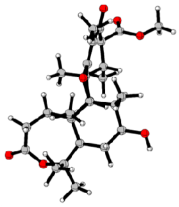
(5*R*,7*S*,8*S*,9*S*,*10S*, 1′*R*,5′*S*,6′*S*)-1-3

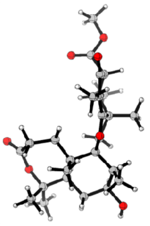
(5*R*,7*S*,8*S*,9*S*,10*S*, 1′*S*,5′*R*,6′*R*)-1-1

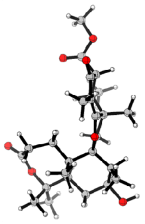
(5*R*,7*S*,8*S*,9*S*,10*S*, 1′*S*,5′*R*,6′*R*)-1-2

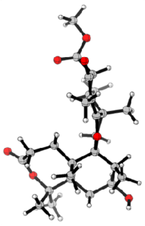
(5*R*,7*S*,8*S*,9*S*,10*S*, 1′*S*,5′*R*,6′*R*)-1-3

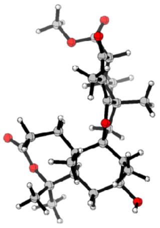
(5*R*,7*S*,8*S*,9*S*,10*S*, 1′*S*,5′*R*,6′*R*)-1-4

Figure S19. Optimized conformers of compound **1**

Table S4. Optimized cartesian coordinates of conformers of (5*R*,7*S*,8*S*,9*S*,*10S*,1′*R*,5′*S*,6′*S*)-**1** at B3LYP/6-311G(d) level in methanol.

	(5*R*,7*S*,8*S*,9*S*,*10S*,1′*R*,5′*S*,6′*S*)-**1-1**			(5*R*,7*S*,8*S*,9*S*,*10S*,1′*R*,5′*S*,6′*S*)-**1-2**			(5*R*,7*S*,8*S*,9*S*,*10S*,1′*R*,5′*S*,6′*S*)-**1-3**		
C	0.68799	0.52586	0.29894	0.68767	0.52835	0.29475	0.61818	0.43499	0.36721
C	2.15318	0.06146	0.63761	2.15302	0.06672	0.63648	2.10634	0.00625	0.65445
C	3.02152	0.4483	-0.6079	3.02012	0.44133	-0.61339	2.95766	0.60449	-0.51682
C	2.92854	1.96497	-0.87491	2.92798	1.95677	-0.89287	2.77968	2.13673	-0.57145
C	1.50008	2.40333	-1.1499	1.49473	2.40154	-1.1664	1.32299	2.52824	-0.80244
C	0.5435	2.044	0.00004	0.53886	2.04306	-0.01644	0.38985	1.96995	0.27405
C	2.11028	-1.46217	0.89164	2.10971	-1.45441	0.90475	2.14338	-1.53772	0.69854
C	3.43078	-2.17377	1.20664	3.43006	-2.16383	1.2251	3.50017	-2.21549	0.92059
C	4.2514	-2.48272	-0.0196	4.24889	-2.48575	0.00096	4.33082	-2.3115	-0.33331
O	4.55282	-1.50065	-0.88979	4.54722	-1.51301	-0.8811	4.59285	-1.20279	-1.05073
C	4.49884	-0.03703	-0.68275	4.49628	-0.04744	-0.68825	4.45918	0.21305	-0.64247

O	4.60497	-3.60643	-0.29973	4.60323	-3.61199	-0.26726	4.72938	-3.36783	-0.77092
C	2.61342	0.73383	1.95404	2.6144	0.75103	1.94603	2.52668	0.51523	2.05484
C	5.42132	0.36687	0.46585	5.42295	0.366	0.45355	5.3549	0.50246	0.56075
C	5.14375	0.43373	-1.99507	5.13727	0.40962	-2.00732	5.08219	0.8945	-1.87021
C	-0.02674	-0.25689	-0.84649	0.02615	-0.26227	-0.84549	0.05239	-0.22492	-0.87845
C	-1.37336	-0.12382	1.10441	1.37358	-0.11593	1.10437	1.40524	-0.42748	1.06155
O	-0.1436	0.2525	1.48568	0.14354	0.26279	1.48349	0.19853	-0.03737	1.49935
C	-1.33818	-0.81121	-0.23412	1.3381	-0.81193	-0.22968	1.32908	-0.93132	-0.35537
C	-2.60628	-0.42333	-1.02275	2.60608	-0.42921	-1.0211	2.61777	-0.51445	-1.09584
C	-3.85259	-0.72411	-0.16956	3.85251	-0.72435	-0.16605	3.8427	-1.00591	-0.29593
C	-3.77544	-0.21958	1.28797	3.77557	-0.21053	1.28817	3.79747	-0.68363	1.21101
C	-2.47542	0.10062	1.85625	2.47553	0.11331	1.85461	2.51914	-0.36183	1.82618
C	-2.43202	0.72695	3.22144	2.43274	0.74849	3.21572	2.51444	0.08861	3.25949
O	-4.81759	-0.06216	1.91708	4.81759	-0.04932	1.9164	4.84736	-0.67408	1.84708
C	-5.09227	-0.10882	-0.7924	5.09215	-0.11306	-0.79299	5.14511	-0.46883	-0.85885
O	-6.10288	-0.98364	-0.85905	6.10232	-0.98885	-0.85546	5.14968	0.87309	-0.87258
O	-5.16266	1.03347	-1.18432	5.16294	1.02697	-1.19116	6.06181	-1.14716	-1.2574
C	-7.34279	-0.4782	-1.39577	7.342	-0.487	-1.39613	6.34772	1.50021	-1.36942
C	-2.69642	-1.08508	-2.3982	2.69615	-1.10051	-2.39192	2.67036	-0.98606	-2.54887
C	-1.23569	-2.33686	-0.01219	1.23592	-2.33612	0.00245	1.13826	-2.46407	-0.33565
C	0.65544	3.00921	1.18531	0.64682	3.01547	1.16451	0.44474	2.7687	1.58147
O	1.53759	3.80777	-1.40713	1.42976	3.78654	-1.50694	1.15747	3.94648	-0.8341
H	1.44283	-1.626	1.73948	1.44281	-1.61006	1.75452	1.48736	-1.8498	1.51298
H	1.6614	-1.98071	0.04497	1.65972	-1.98021	0.06319	1.72078	-1.95914	-0.21298
H	2.55724	-0.05027	-1.46687	2.55257	-0.06192	-1.46779	2.52323	0.20569	-1.44127
H	1.16517	1.89055	-2.06016	1.15902	1.89747	-2.07577	1.02261	2.11887	-1.77532
H	-0.48065	2.15623	-0.37914	0.48135	2.1571	-0.40072	0.63496	2.08286	-0.09936
H	0.57975	-1.05554	-1.26375	0.58009	-1.06446	-1.25624	0.59842	-0.92288	-1.39728
H	-0.25389	0.41668	-1.67003	0.25283	0.40533	-1.67416	0.3197	0.54102	-1.60349
H	-4.01531	-1.80379	-0.11418	4.01537	-1.80364	-0.10369	3.92181	-2.09304	-0.38083
H	-2.57328	0.66207	-1.16266	2.57303	0.65516	-1.16847	2.64344	0.57941	-1.08611
H	3.33536	2.5425	-0.04207	3.34658	2.53344	-0.06308	3.14885	2.61709	0.33676
H	3.51118	2.23949	-1.7511	3.50759	2.22413	-1.77327	3.35651	2.55502	-1.39525
H	3.21456	-3.13912	1.66325	3.21362	-3.12442	1.69153	3.33609	-3.24365	1.24147
H	4.03285	-1.62672	1.93248	4.03362	-1.61035	1.9448	4.0747	-1.73985	1.71545
H	3.06649	1.70959	1.80722	3.35593	0.15293	2.46955	2.93019	1.52326	2.04676
H	1.77142	0.85327	2.63222	3.05857	1.72988	1.79188	1.67704	0.4992	2.73372
H	3.3467	0.12569	2.47782	1.77483	0.86678	2.62797	3.28797	-0.1214	2.49911
H	6.43778	0.03613	0.24469	6.43792	0.03049	0.23284	5.30992	1.56452	0.80716
H	5.13711	-0.0431	1.42862	5.1406	-0.03351	1.42126	6.38872	0.26177	0.30629
H	5.43719	1.45361	0.56218	5.44224	1.45363	0.53903	5.09168	-0.05203	1.45473
H	6.05188	-0.14592	-2.16074	5.41498	1.46216	-1.97873	5.30458	1.94356	-1.68118
H	4.4758	0.27533	-2.84425	6.04421	-0.17258	-2.17035	6.01933	0.39038	-2.10618
H	5.42018	1.4862	-1.955	4.46629	0.24352	-2.85262	4.42564	0.82237	-2.73979

H	-3.07389	1.61026	3.26764	3.06474	1.63942	3.25236	1.51613	0.39043	3.57345
H	-2.79824	0.03067	3.98151	2.81134	0.0615	3.97806	3.19713	0.92927	3.40707
H	-1.41676	1.01828	3.48738	1.41587	1.03028	3.48576	2.85381	-0.71345	3.92128
H	-7.19652	-0.12315	-2.41537	7.7153	0.33043	-0.77994	6.16289	2.56877	-1.30731
H	-8.03017	-1.31894	-1.37763	7.19503	-0.1379	-2.41767	6.53094	1.20174	-2.40124
H	-7.71529	0.33572	-0.77446	8.02908	-1.32788	-1.3736	7.20063	1.22188	-0.75092
H	-3.53034	-0.67218	-2.97015	1.78724	-0.93578	-2.97501	2.75915	-2.07244	-2.61926
H	-1.78757	-0.91609	-2.98018	2.85047	-2.17843	-2.30554	3.52913	-0.5514	-3.06592
H	-2.85044	-2.16361	-2.31935	3.52985	-0.69132	-2.96683	1.77436	-0.68397	-3.09574
H	-0.42211	-2.57686	0.67083	2.15083	-2.7441	0.43237	2.02659	-2.98065	0.02784
H	-2.15035	-2.74807	0.4152	1.0386	-2.85115	-0.93942	0.9125	-2.83131	-1.33838
H	-1.03843	-2.84548	-0.95757	0.42264	-2.57176	0.68736	0.31194	-2.74481	0.31552
H	1.67977	3.19653	1.50004	1.66971	3.21657	1.48063	0.01569	3.74193	1.41308
H	0.09553	2.63644	2.04227	0.09951	2.64099	2.02818	1.45494	2.94929	1.94235
H	0.22882	3.97111	0.89947	0.196	3.96842	0.88171	0.11321	2.26031	2.36705
H	0.65802	4.08913	-1.68579	1.81297	4.2986	-0.78452	1.66433	4.28725	-1.58029

Table S5. Optimized cartesian coordinates of conformers of (5*R*,7*S*,8*S*,9*S*,10*S*,1′*S*,5′*R*,6′*R*)-**1** at B3LYP/6-311G(d) level in methanol**.**

	(5*R*,7*S*,8*S*,9*S*,10*S*,1′*S*,5′*R*,6′*R*)-**1-1**			(5*R*,7*S*,8*S*,9*S*,10*S*,1′*S*,5′*R*,6′*R*)-**1-2**			(5*R*,7*S*,8*S*,9*S*,10*S*,1′*S*,5′*R*,6′*R*)-**1-3**			(5*R*,7*S*,8*S*,9*S*,10*S*,1′*S*,5′*R*,6′*R*)-**1-4**		
C	-0.98591	-1.18765	0.08144	-0.98574	-1.18668	0.08447	-0.95854	-1.21887	0.0872	-0.98678	-1.18692	0.07967
C	-1.86779	0.0062	0.63659	-1.86899	0.00679	0.6367	-1.78257	0.01045	0.65208	-1.87019	0.00453	0.63563
C	-2.93023	0.29938	-0.47551	-2.92819	0.29874	-0.47903	-2.85255	0.33906	-0.4429	-2.92484	0.30443	-0.48209
C	-3.76269	-0.97192	-0.74944	-3.76309	-0.96888	-0.75767	-3.74412	-0.89641	-0.69359	-3.76167	-0.96126	-0.76857
C	-2.89112	-2.13253	-1.22157	-2.89833	-2.12772	-1.22479	-2.93705	-2.10047	-1.1693	-2.89812	-2.12953	-1.23463
C	-1.77299	-2.48151	-0.23497	-1.77495	-2.47857	-0.23211	-1.80017	-2.48258	-0.20453	-1.77236	-2.47856	-0.24491
C	-0.91477	1.19433	0.91736	-0.917	1.19523	0.91952	-0.77933	1.16187	0.90933	-0.91745	1.18989	0.92863
C	-1.53897	2.51446	1.38343	-1.54282	2.51528	1.38353	-1.34328	2.50766	1.37882	-1.54275	2.50868	1.39681
C	-2.12808	3.32856	0.26046	-2.12955	3.32868	0.25882	-1.91745	3.33912	0.26089	-2.12277	3.32943	0.27393
O	-3.06089	2.78699	-0.54483	-3.0595	2.78605	-0.54899	-2.88104	2.82921	-0.52875	-3.04907	2.79241	-0.54212
C	-3.84735	1.5502	-0.34563	-3.84632	1.54935	-0.35158	-3.71501	1.62772	-0.30823	-3.84006	1.55704	-0.35375
O	-1.75384	4.45164	0.00539	-1.75544	4.45198	0.00449	-1.50433	4.44582	-0.00525	-1.74612	4.45367	0.02808
C	-2.48814	-0.39398	1.99667	-2.49285	-0.39326	1.99517	-2.39248	-0.3595	2.02516	-2.4989	-0.40257	1.98952
C	-4.69876	1.65886	0.91812	-4.70109	1.65811	0.9099	-4.54159	1.78045	0.9674	-4.70036	1.66281	0.90424
C	-4.78705	1.64455	-1.55724	-4.78306	1.6431	-1.56555	-4.66839	1.75205	-1.50639	-4.77058	1.65796	-1.57192
C	-0.09898	-0.80621	-1.14177	-0.09853	-0.80644	-1.13891	-0.08783	-0.87875	-1.15936	-0.09931	-0.80178	-1.14159
C	1.23426	-1.19436	0.77231	1.2346	-1.19223	0.77577	1.27561	-1.30015	0.72683	1.23332	-1.19612	0.77175
O	-0.01455	-1.50886	1.14176	-0.01421	-1.50602	1.14596	0.02517	-1.56862	1.12756	-0.01606	-1.51011	1.14086
C	1.35527	-1.12917	-0.72355	1.35552	-1.12987	-0.72033	1.36336	-1.25187	-0.77286	1.35431	-1.12885	-0.72418
C	2.37373	-0.03566	-1.10028	2.37439	-0.03753	-1.09939	2.40918	-0.19522	-1.18055	2.374	-0.03594	-1.09948
C	3.69126	-0.28097	-0.3425	3.69189	-0.28143	-0.34113	3.7366	-0.4893	-0.4521	3.69127	-0.28299	-0.34183
C	3.51814	-0.53212	1.17109	3.51873	-0.52943	1.17298	3.58998	-0.71605	1.06567	3.5177	-0.53581	1.17132
C	2.22471	-0.97075	1.66716	2.22519	-0.9668	1.66998	2.29308	-1.10148	1.59676	2.22381	-0.97427	1.66679

C	2.03642	-1.09692	3.15254	2.03701	-1.08947	3.15568	2.13535	-1.20644	3.08726	2.03565	-1.10174	3.15207
O	4.46905	-0.32833	1.92072	4.46957	-0.32414	1.92223	4.56696	-0.54461	1.78949	4.46842	-0.3335	1.92147
C	4.632	0.89927	-0.49872	4.63255	0.89862	-0.4997	4.765	0.60353	-0.6737	4.63218	0.89737	-0.49656
O	5.86949	0.5097	-0.82794	5.87018	0.50851	-0.82777	4.29283	1.79775	-0.28382	5.8693	0.50833	-0.82782
O	4.30702	2.05455	-0.34696	4.30734	2.05417	-0.35056	5.86358	0.4384	-1.14803	4.30745	2.05238	-0.34226
C	6.85039	1.55966	-0.95544	6.85091	1.55841	-0.95739	5.18997	2.91545	-0.42845	6.8499	1.55866	-0.95515
C	2.60048	0.09143	-2.60685	2.60113	0.08619	-2.60625	2.60459	-0.07867	-2.69184	2.60096	0.09265	-2.6059
C	1.76333	-2.52518	-1.24806	1.76305	-2.52695	-1.24233	1.70901	-2.66448	-1.29613	1.7606	-2.5244	-1.25106
C	-2.26147	-3.30778	0.96087	-2.26804	-3.30483	0.96106	-2.28916	-3.28603	1.00707	-2.2608	-3.31229	0.94623
O	-3.66324	-3.31407	-1.43916	-3.77489	-3.23028	-1.46047	-3.78052	-3.21311	-1.46692	-3.69218	-3.26957	-1.5634
H	-0.22358	0.8773	1.69979	-0.22778	0.87865	1.70387	-0.08712	0.82203	1.6811	-0.23215	0.86799	1.71415
H	-0.29709	1.42338	0.04885	-0.29711	1.4241	0.05248	-0.16902	1.36201	0.02844	-0.29349	1.42176	0.06534
H	-2.36541	0.50721	-1.39174	-2.36063	0.50776	-1.39309	-2.29673	0.51617	-1.37076	-2.3541	0.514	-1.39398
H	-2.43358	-1.8329	-2.17317	-2.44036	-1.83522	-2.17813	-2.50048	-1.84292	-2.13736	-2.4475	-1.84645	-2.18903
H	-1.07109	-3.12457	-0.77466	-1.06745	-3.12095	-0.76813	-1.13753	-3.15492	-0.75735	-1.07111	-3.1198	-0.78692
H	-0.19688	0.24107	-1.41429	-0.19573	0.24095	-1.41121	-0.1534	0.16992	-1.43617	-0.19457	0.24722	-1.40834
H	-0.38223	-1.37963	-2.0211	-0.38203	-1.37939	-2.01854	-0.41225	-1.4463	-2.02852	-0.3831	-1.3699	-2.02437
H	4.1978	-1.16148	-0.74715	4.19854	-1.16269	-0.74397	4.18729	-1.39679	-0.86337	4.19762	-1.16312	-0.74751
H	1.97251	0.91376	-0.73124	1.97344	0.9128	-0.7324	2.04572	0.76607	-0.80528	1.97367	0.91345	-0.7294
H	-4.3327	-1.27664	0.13024	-4.33633	-1.27057	0.1214	-4.31213	-1.1567	0.20382	-4.34218	-1.25262	0.11112
H	-4.49203	-0.78291	-1.53633	-4.48821	-0.78709	-1.54752	-4.47793	-0.69287	-1.47	-4.48252	-0.77698	-1.56175
H	-0.75975	3.13868	1.81932	-0.76494	3.13997	1.82114	-0.53321	3.10249	1.79928	-0.76546	3.12938	1.84105
H	-2.28038	2.36392	2.16793	-2.28614	2.36472	2.16624	-2.0773	2.38996	2.17573	-2.28961	2.35568	2.17568
H	-2.67523	0.47749	2.61957	-1.80628	-1.02689	2.55223	-2.5441	0.52203	2.64346	-2.69684	0.46602	2.61293
H	-3.43313	-0.92098	1.90722	-2.68097	0.4782	2.61779	-3.35429	-0.85888	1.95667	-3.44017	-0.93508	1.89228
H	-1.79981	-1.02716	2.552	-3.43802	-0.91953	1.90326	-1.71381	-1.00785	2.57508	-1.81068	-1.0324	2.54898
H	-4.12667	1.74978	1.83473	-5.33426	0.77421	1.00245	-5.21356	0.92739	1.07491	-5.33757	0.78109	0.98992
H	-5.33172	0.77497	1.01231	-5.35333	2.52964	0.82922	-5.1543	2.68078	0.89398	-5.34897	2.5371	0.82457
H	-5.35106	2.53052	0.83941	-4.13155	1.74926	1.82809	-3.95267	1.84844	1.8753	-4.13486	1.74765	1.82552
H	-5.61851	0.94561	-1.47979	-5.19499	2.6515	-1.60663	-5.04011	2.77593	-1.54716	-5.18076	2.66715	-1.61038
H	-5.1993	2.65286	-1.59677	-4.24507	1.4541	-2.49682	-4.15476	1.53586	-2.44544	-4.22821	1.47246	-2.50132
H	-4.25125	1.45646	-2.48999	-5.61453	0.94411	-1.48974	-5.52595	1.08771	-1.41184	-5.60357	0.95996	-1.50362
H	2.35165	-0.18388	3.66351	0.99511	-1.28791	3.40429	2.72307	-2.03818	3.48693	2.64697	-1.91347	3.55735
H	0.9942	-1.29422	3.40069	2.64584	-1.90195	3.56328	2.49486	-0.30023	3.58089	2.34629	-0.18728	3.66338
H	2.64376	-1.91161	3.55798	2.35055	-0.17451	3.66428	1.09301	-1.36402	3.36194	0.99434	-1.30404	3.39996
H	6.96348	2.08349	-0.00672	7.77591	1.0585	-1.22973	6.08387	2.75904	0.17483	7.77429	1.05974	-1.23133
H	7.77515	1.06019	-1.22941	6.55121	2.26183	-1.73358	5.4711	3.0421	-1.47355	6.54871	2.2652	-1.72791
H	6.55053	2.26505	-1.72978	6.96345	2.08458	-0.00989	4.63586	3.77983	-0.07394	6.96437	2.08102	-0.00579
H	1.6541	0.20756	-3.14012	3.21101	0.96329	-2.83286	1.65052	0.06958	-3.20284	3.21148	0.97008	-2.82948
H	3.11313	-0.78244	-3.01527	1.6548	0.20147	-3.1398	3.07764	-0.96993	-3.11013	1.65476	0.2104	-3.13911
H	3.21009	0.96922	-2.83153	3.11353	-0.78874	-3.01275	3.23975	0.77645	-2.93441	3.11274	-0.78127	-3.01528
H	1.09194	-3.2931	-0.86227	2.77546	-2.79572	-0.93988	1.63446	-2.69551	-2.38428	2.77283	-2.79485	-0.94947
H	1.70953	-2.55262	-2.33751	1.09307	-3.29452	-0.8533	2.71485	-2.97188	-1.009	1.09004	-3.29285	-0.86492

H	2.77505	-2.79519	-0.94442	1.70743	-2.55703	-2.33162	1.0162	-3.40425	-0.89359	1.70501	-2.55062	-2.34043
H	-2.5105	-4.31024	0.61402	-1.48864	-3.38964	1.71822	-2.55918	-4.29204	0.68107	-3.15635	-2.91979	1.42597
H	-3.15061	-2.90277	1.43885	-2.51316	-4.31074	0.61906	-3.15826	-2.85669	1.50368	-1.48381	-3.39199	1.7057
H	-1.47684	-3.39385	1.71248	-3.16188	-2.90164	1.43152	-1.49737	-3.3861	1.74862	-2.48661	-4.32359	0.60341
H	-4.30202	-3.12645	-2.13653	-3.26274	-3.94748	-1.85269	-4.27722	-3.44312	-0.67229	-4.20334	-3.52275	-0.7851

Table S6. Conformational analysis of the optimized isomers of **2** in chloroform

Conformations	*G* (hartree)	*ΔG* (kcal/mol)	Boltzmann distributions (%)
**2-1**	-1579.501745	0	79.8
**2-2**	-1579.499694	1.287021779	9.1
**2-3**	-1579.498935	1.763301414	4.1
**2-4**	-1579.498698	1.912021142	3.2
**2-5**	-1579.498324	2.146709657	2.1
**2-6**	-1579.498156	2.252131237	1.7

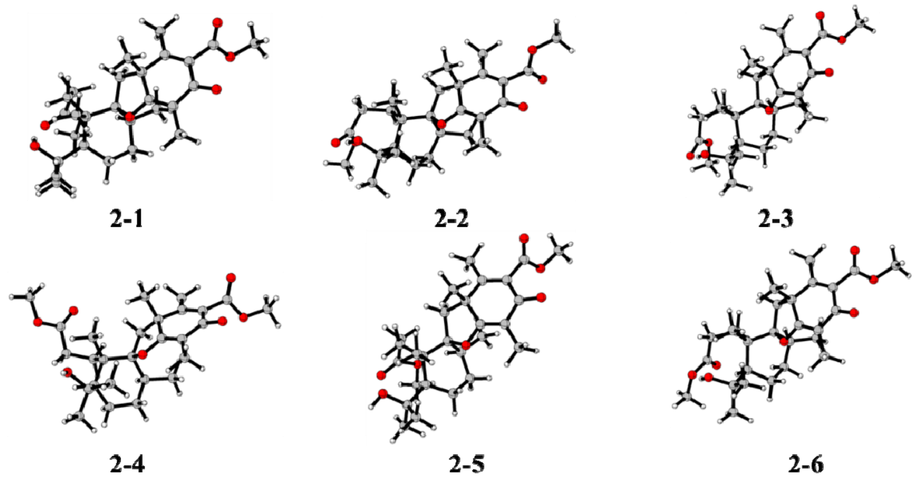

Figure S20. Optimized conformers of compound **2**

Table S7. Optimized cartesian coordinates of conformers of **2** at B3LYP/6-311G(d) level in chloroform**.**

	**2-1**			**2-2**			**2-3**		
C	-2.4531	0.67803	1.73141	-2.45328	-1.79266	-0.30502	-2.46501	-1.76469	-0.40323
C	-2.62636	2.09034	1.15729	-3.77853	-2.03837	-1.0599	-3.83275	-1.94836	-1.0973
C	-4.16583	-3.0149	0.08247	-2.26283	2.89103	-1.12741	-2.27839	2.96692	-0.8817
C	-4.2622	-1.49621	-0.06957	-3.31256	1.89724	-0.63639	-3.30969	1.94537	-0.4089
C	-2.89413	-0.78278	-0.36096	-2.74305	0.70314	0.20319	-2.70742	0.68736	0.30525
C	-1.91214	-0.4793	0.83813	-1.80235	-0.39037	-0.49131	-1.81505	-0.35446	-0.52002
C	-2.13347	-1.47617	-1.50246	-2.19056	1.18811	1.55685	-2.08008	1.06355	1.66103
C	-1.13506	-0.50478	-2.14241	-1.70203	0.01541	2.39612	-1.55479	-0.17228	2.37896
C	-0.58569	0.5277	-1.1388	-0.51275	-0.71559	1.74207	-0.40728	-0.85288	1.60687

C	-0.48078	-0.12079	0.26144	-0.40661	-0.44561	0.21633	-0.38284	-0.4666	0.10295
C	0.446	0.66085	1.22796	0.63613	-1.42602	-0.39993	0.61836	-1.3985	-0.6431
C	1.79569	-0.1039	1.23117	1.86014	-0.55819	-0.78646	1.82648	-0.50716	-1.0259
C	1.57045	-1.1751	0.20554	1.52634	0.77016	-0.17017	1.53321	0.77128	-0.29407
O	0.26109	-1.39905	0.04376	0.20558	0.89249	0.02083	0.22547	0.88111	-0.02296
C	3.06747	0.67168	0.98848	3.22767	-1.05172	-0.37434	3.21058	-1.03561	-0.72364
C	4.04689	0.06687	0.29299	4.13557	-0.13976	0.01081	4.14577	-0.1563	-0.32311
C	3.89742	-1.27942	-0.33511	3.83192	1.3111	0.16803	3.85754	1.28547	-0.06222
C	2.55099	-1.83192	-0.44726	2.43086	1.7169	0.15472	2.45984	1.68978	0.04636
C	2.31449	-2.98434	-1.3782	2.0687	3.07942	0.66809	2.13734	3.01497	0.67164
O	4.88173	-1.83756	-0.82652	4.74698	2.10118	0.41744	4.7885	2.06557	0.15393
C	5.39319	0.6946	0.10153	5.54281	-0.51385	0.3636	5.57706	-0.55009	-0.12416
O	5.56919	1.07662	-1.17044	6.32278	-0.58059	-0.72522	5.90425	-0.53773	1.17478
O	6.22085	0.84698	0.97092	5.93243	-0.72857	1.48711	6.34691	-0.84632	-1.00947
C	-5.23481	-1.17555	-1.21934	-4.35819	2.65974	0.19981	-4.29939	2.64862	0.53973
O	-4.87189	-1.02789	1.14871	-3.99742	1.42202	-1.81428	-4.06361	1.56475	-1.57908
C	-3.82155	2.25791	0.25342	-5.00919	-1.49436	-0.37808	-5.01781	-1.44755	-0.30964
O	-3.55025	3.03029	-0.80262	-5.22494	-2.08923	0.79945	-5.1687	-2.11903	0.83648
O	-4.92207	1.78045	0.45878	-5.74055	-0.62619	-0.81932	-5.7671	-0.54917	-0.6482
C	-4.64091	3.28769	-1.71443	-6.35364	-1.61713	1.56525	-6.24493	-1.69272	1.69865
C	0.69754	1.15873	-1.68915	0.78842	-0.40562	2.49275	0.9347	-0.60381	2.30687
C	1.97785	-0.81847	2.6077	1.93664	-0.36836	-2.33388	1.82372	-0.20068	-2.55635
C	3.19079	2.0254	1.61718	3.52291	-2.51339	-0.52355	3.47339	-2.4872	-0.98456
C	6.86097	1.63074	-1.49198	7.70846	-0.90963	-0.49004	7.28192	-0.83081	1.48348
C	-1.76708	-1.67449	1.79404	-1.57554	-0.17633	-1.9992	-1.67079	-0.02886	-2.018
H	-1.81015	0.76763	2.60675	-2.6224	-2.00342	0.75098	-2.57605	-2.05487	0.64153
H	-3.40985	0.34851	2.12485	-1.74923	-2.54988	-0.65647	-1.78757	-2.49516	-0.85066
H	-1.73595	2.47269	0.66212	-3.92046	-3.11977	-1.13637	-3.98895	-3.02079	-1.24222
H	-2.81229	2.76424	2.00169	-3.74958	-1.64106	-2.07211	-3.85734	-1.48009	-2.07888
H	-3.77151	-3.48039	-0.82142	-2.76092	3.70915	-1.65357	-2.79603	3.82897	-1.30975
H	-5.16742	-3.41403	0.25768	-1.55088	2.4351	-1.80966	-1.61465	2.56124	-1.64008
H	-3.53799	-3.30918	0.91973	-1.7058	3.31481	-0.29131	-1.66679	3.31714	-0.04974
H	-3.18401	0.1919	-0.76296	-3.6435	0.15382	0.48541	-3.59692	0.12302	0.59207
H	-1.61128	-2.35237	-1.11711	-1.38416	1.90842	1.41669	-1.27768	1.79088	1.53452
H	-2.82332	-1.83852	-2.2652	-2.98373	1.69872	2.10314	-2.83955	1.53144	2.28755
H	-1.61439	0.04185	-2.96082	-2.53558	-0.67788	2.55044	-2.38332	-0.8735	2.52395
H	-0.30346	-1.0607	-2.58324	-1.41415	0.36523	3.39112	-1.21156	0.09832	3.38108
H	-1.31906	1.33213	-1.04795	-0.68212	-1.79341	1.82268	-0.57828	-1.93345	1.61408
H	0.55063	1.6945	0.91003	0.90153	-2.18698	0.33052	0.9162	-2.21474	0.01087
H	0.05079	0.68474	2.23795	0.25121	-1.94882	-1.27043	0.18401	-1.85095	-1.52958
H	1.24969	-3.19752	-1.47111	0.98877	3.22465	0.66385	1.05997	3.16008	0.74682
H	2.71701	-2.77538	-2.37329	2.43529	3.2278	1.68836	2.56957	3.09515	1.67369
H	2.81468	-3.88851	-1.01898	2.52336	3.8612	0.05339	2.55497	3.83588	0.08222
H	-6.19762	-1.65095	-1.01958	-5.08888	1.97184	0.63573	-5.01144	1.93388	0.96318

H	-4.87735	-1.53094	-2.18707	-4.89219	3.34384	-0.4624	-4.86276	3.38414	-0.03764
H	-5.39795	-0.09804	-1.29106	-3.91595	3.24729	1.00515	-3.80675	3.17073	1.36076
H	-5.07104	-0.08538	1.02977	-4.70205	0.81364	-1.53761	-4.75734	0.94071	-1.3102
H	-4.96321	2.35805	-2.18189	-6.36478	-2.22499	2.46499	-6.21451	-2.36561	2.55038
H	-5.47589	3.7428	-1.18425	-6.22182	-0.56475	1.81472	-6.08245	-0.66344	2.01738
H	-4.2383	3.96906	-2.45784	-7.2747	-1.74837	0.99941	-7.19851	-1.77067	1.17885
H	1.50667	0.43259	-1.78115	0.70831	-0.77037	3.51917	1.12799	0.46411	2.4218
H	1.05774	1.9947	-1.08726	0.97305	0.66916	2.53566	1.78699	-1.04198	1.78225
H	0.49897	1.55031	-2.69023	1.66879	-0.87767	2.05061	0.90836	-1.04634	3.30513
H	1.12539	-1.46737	2.80959	2.76242	0.29333	-2.59421	1.94728	-1.12677	-3.12083
H	2.88544	-1.4215	2.61182	2.09223	-1.33373	-2.81918	0.88368	0.25474	-2.85853
H	2.04033	-0.07852	3.40782	1.01291	0.05659	-2.71894	2.63881	0.47684	-2.80953
H	2.60556	2.75951	1.05395	3.10065	-3.07419	0.31647	3.01656	-2.80784	-1.92416
H	2.79387	2.02474	2.6354	3.07129	-2.91957	-1.43151	4.53701	-2.71269	-1.02909
H	4.22189	2.37143	1.6535	4.59275	-2.71748	-0.55622	3.02751	-3.10093	-0.19541
H	7.0443	2.53103	-0.90609	8.17301	-0.92182	-1.47172	7.93598	-0.10056	1.00751
H	7.64286	0.89852	-1.29178	8.17041	-0.15398	0.14461	7.35658	-0.76334	2.565
H	6.81926	1.86573	-2.55163	7.78914	-1.88656	-0.01435	7.54256	-1.83238	1.1425
H	-2.71409	-1.85829	2.29782	-1.17731	-1.08029	-2.46384	-2.61532	0.28149	-2.44687
H	-1.4557	-2.58134	1.28063	-2.49616	0.09003	-2.50316	-0.96778	0.78342	-2.17749
H	-1.0222	-1.46097	2.5636	-0.8705	0.62978	-2.17966	-1.31038	-0.89791	-2.57161

		**2-4**			**2-5**			**2-6**	
C	2.67694	0.84113	1.49073	-2.49873	0.66424	1.75879	-2.40944	-1.88601	-0.11739
C	4.0359	1.47295	1.16878	-2.54102	2.12807	1.27461	-3.81075	-2.1227	-0.71628
C	1.94671	-2.94887	-1.42452	-4.12651	-3.04819	-0.15405	-2.30558	2.75548	-1.12098
C	3.17179	-2.41695	-0.68016	-4.27563	-1.52322	-0.05441	-3.31008	1.79578	-0.48861
C	2.93041	-1.47561	0.54834	-2.95974	-0.72797	-0.34421	-2.68405	0.62365	0.33827
C	2.10408	-0.12846	0.40115	-1.95029	-0.4724	0.83998	-1.7875	-0.4846	-0.38985
C	2.51156	-2.23129	1.82895	-2.23451	-1.31947	-1.56236	-2.04774	1.13986	1.64267
C	1.00865	-2.40226	1.99827	-1.24203	-0.3121	-2.15142	-1.48581	-0.01	2.46742
C	0.28341	-1.05666	2.00643	-0.63788	0.63002	-1.09252	-0.33174	-0.73483	1.74769
C	0.56934	-0.27536	0.70256	-0.52789	-0.10406	0.26134	-0.34303	-0.50483	0.21144
C	0.21424	1.07153	0.67982	0.41584	0.60323	1.26792	0.66421	-1.48905	-0.45544
C	1.35558	0.87541	-0.34902	1.74886	-0.18831	1.22814	1.84331	-0.61787	-0.95733
C	1.2774	-0.59271	-0.64736	1.50627	-1.18849	0.1361	1.5371	0.72325	-0.35402
O	0.04951	-1.06272	-0.3884	0.19461	-1.37755	-0.04392	0.23324	0.83439	-0.06485
C	2.74559	1.31465	0.04421	3.03483	0.5785	1.03542	3.24576	-1.08344	-0.63589
C	3.78296	0.56431	-0.36732	4.0053	0.00112	0.30488	4.1691	-0.14788	-0.35288
C	3.62217	-0.72572	-1.10073	3.83347	-1.30203	-0.40387	3.85356	1.30661	-0.23458
C	2.29707	-1.33646	-1.12234	2.47791	-1.82079	-0.55405	2.4498	1.69046	-0.13098
C	2.17087	-2.77683	-1.52241	2.22429	-2.90767	-1.55667	2.11052	3.06579	0.3633
O	4.61289	-1.28448	-1.57918	4.80979	-1.84841	-0.92394	4.77096	2.12407	-0.124
C	5.20466	0.97953	-0.14484	5.36183	0.61689	0.15232	5.61419	-0.48737	-0.15289

O	5.79969	0.19714	0.76544	5.55352	1.05376	-1.09958	5.96968	-0.34529	1.13092
O	5.75572	1.90068	-0.70344	6.18496	0.71813	1.03362	6.37157	-0.84651	-1.02548
C	4.04506	-3.61573	-0.2646	-5.35385	-1.04674	-1.03919	-4.25924	2.61231	0.40921
O	3.97413	-1.62515	-1.58941	-4.732	-1.19751	1.27601	-4.11114	1.27764	-1.57457
C	3.93482	2.7094	0.30513	-3.65115	2.42755	0.29485	-4.93497	-1.71662	0.20598
O	5.00025	2.83499	-0.5029	-3.18106	2.91306	-0.87117	-5.79881	-0.85458	-0.38291
O	3.03189	3.51587	0.34265	-4.83038	2.27135	0.51617	-5.07881	-2.11181	1.33838
C	5.02426	4.00502	-1.34432	-4.16413	3.21161	-1.88138	-6.94	-0.45683	0.41016
C	1.20586	-1.2737	2.30282	0.65723	1.25003	-1.62797	1.0139	-0.37805	2.3916
C	1.02405	1.63477	-1.67267	1.91749	-0.98949	2.55754	1.80132	-0.47281	-2.51089
C	2.8819	2.60907	0.78596	3.17882	1.88923	1.74597	3.53753	-2.54841	-0.75111
C	7.19598	0.46097	1.01007	6.85525	1.60353	-1.3858	7.36118	-0.5748	1.43159
C	2.28143	0.51917	-0.98106	-1.79474	-1.70016	1.75435	-1.67954	-0.31586	-1.91699
H	2.76693	0.31402	2.43847	-1.92893	0.6592	2.68953	-2.4673	-2.08858	0.95244
H	1.98223	1.66016	1.67375	-3.51138	0.3755	2.0281	-1.74378	-2.64433	-0.53414
H	4.73783	0.77351	0.71492	-1.59046	2.47831	0.87833	-3.93949	-3.19824	-0.86879
H	4.50042	1.80201	2.10521	-2.75751	2.74169	2.15449	-3.93096	-1.64848	-1.68755
H	1.35327	-2.15018	-1.85646	-3.87496	-3.36964	-1.16561	-2.84312	3.58354	-1.5896
H	1.29928	-3.5298	-0.76693	-5.07881	-3.51883	0.10887	-1.69867	2.27508	-1.88248
H	2.27678	-3.61371	-2.23019	-3.36748	-3.42896	0.52644	-1.63684	3.16894	-0.36581
H	3.95341	-1.13471	0.73238	-3.30412	0.26009	-0.65698	-3.56606	0.08132	0.68695
H	3.00444	-3.20041	1.87804	-1.71111	-2.22992	-1.26846	-1.26331	1.86824	1.43377
H	2.88947	-1.68303	2.69664	-2.95121	-1.60902	-2.33286	-2.80861	1.65061	2.23287
H	0.80378	-2.92421	2.93841	-1.73822	0.30584	-2.90679	-2.29637	-0.71177	2.68904
H	0.59028	-3.02509	1.20285	-0.43836	-0.84607	-2.66572	-1.13756	0.36242	3.4345
H	0.69753	-0.44623	2.81756	-1.34056	1.44853	-0.94056	-0.47159	-1.81298	1.86952
H	0.59375	1.29822	1.67339	0.54776	1.64834	1.00318	0.99454	-2.22532	0.27367
H	0.41244	1.90881	0.38879	0.01454	0.58548	2.27639	0.22192	-2.04158	-1.2791
H	1.16461	-3.14751	-1.33018	2.6442	-2.64861	-2.53262	2.61134	3.27872	1.31214
H	2.88497	-3.3997	-0.97663	2.69732	-3.84492	-1.24878	2.44139	3.82977	-0.34611
H	2.38553	-2.9102	-2.5869	1.15569	-3.08737	-1.67395	1.03564	3.17526	0.50532
H	4.88936	-3.29599	0.35091	-5.05905	-1.19276	-2.08031	-4.93844	1.96285	0.96964
H	4.44732	-4.0859	-1.16642	-5.55485	0.01343	-0.87704	-4.86158	3.25981	-0.23048
H	3.48724	-4.38285	0.27476	-6.28407	-1.60146	-0.88065	-3.72837	3.24298	1.12326
H	4.12871	-2.15731	-2.37926	-5.59666	-1.60916	1.39424	-4.78041	0.68378	-1.20422
H	4.17143	3.99783	-2.02286	-4.88807	3.93321	-1.50506	-7.54856	-1.32785	0.64751
H	5.00016	4.90991	-0.73782	-3.60585	3.6271	-2.71536	-6.6108	0.02277	1.33092
H	5.95481	3.94266	-1.90132	-4.67966	2.30024	-2.18412	-7.49374	0.24122	-0.2106
H	1.79828	-0.35729	2.27653	1.06249	2.02856	-0.97921	1.1779	0.70091	2.39182
H	1.31402	-1.69589	3.30474	0.45308	1.71784	-2.59478	1.86961	-0.84474	1.89817
H	1.65298	-1.97867	1.60034	1.4362	0.50284	-1.78869	1.01738	-0.71532	3.43048
H	0.07675	1.29032	-2.08269	2.81651	-1.60445	2.52487	0.84677	-0.06726	-2.83735
H	1.80653	1.46825	-2.41276	1.98947	-0.3014	3.40194	2.59763	0.18812	-2.85229
H	0.94256	2.70591	-1.47873	1.05607	-1.63742	2.71835	1.93017	-1.45073	-2.97852

H	2.58715	2.47985	1.8323	2.61551	2.66819	1.22173	3.05969	-2.97764	-1.63518
H	2.22155	3.37312	0.36832	2.76987	1.83611	2.758	4.60485	-2.75323	-0.80807
H	3.90054	2.99149	0.76592	4.21639	2.21012	1.81357	3.13378	-3.08435	0.11379
H	7.33043	1.47495	1.38576	7.62567	0.8529	-1.21088	7.98473	0.12474	0.87527
H	7.76815	0.33252	0.09151	6.82629	1.88465	-2.43461	7.45765	-0.40651	2.50032
H	7.50154	-0.2671	1.75599	7.04514	2.4752	-0.76004	7.64106	-1.59669	1.1771
H	3.33225	0.59436	-1.24237	-2.7429	-1.9219	2.23735	-2.63602	-0.06153	-2.35689
H	1.8117	-0.07107	-1.7626	-1.45963	-2.57869	1.20708	-0.98858	0.48042	-2.17629
H	1.85428	1.52051	-1.00277	-1.0595	-1.50295	2.53717	-1.32138	-1.23493	-2.38425

Table S8. α-Glucosidase and acetylcholinesterase inhibitory activities of all compounds (IC_{50}, μM)

Compounds	α-glucosidase	acetylcholinesterase
**1**	>200	>200
**2**	>200	>200
**3**	>200	>200
**4**	>200	>200
**5**	>200	>200
**6**	>200	>200
**7**	>200	>200
**8**	>200	>200
**9**	104.1	>200
**10**	111.3	>200
**11**	>200	>200
**12**	>200	>200
**13**	>200	>200
**14**	>200	>200
**15**	>200	>200
**16**	>200	>200
**17**	>200	>200
**18**	>200	>200
**Acarbose**	304.6	
**Genistein**	19.95	
**Tacrine**		Undetermined

Table S9. Antibacterial activities of all compounds MIC (μg/mL)

Compounds	*Staphylococcus aureus*	*Bacillus subtilis*	*Escherichia coli*	*Listeria monocytogenes*
**1**	>128	>128	>128	>128
**2**	>128	>128	>128	>128
**3**	>128	>128	>128	>128
**4**	>128	>128	>128	>128
**5**	128	>128	>128	>128
**6**	>128	>128	>128	>128
**7**	>128	>128	>128	>128
**8**	>128	>128	>128	>128
**9**	>128	>128	>128	>128
**10**	>128	>128	>128	>128
**11**	>128	>128	>128	>128
**12**	32	16	>128	>128
**13**	>128	>128	>128	>128
**14**	>128	>128	>128	>128

**15**	>128	>128	>128	>128
**16**	>128	>128	>128	>128
**17**	>128	>128	>128	>128
**18**	>128	>128	>128	>128
**Ampicillin**	<1	<1	<1	<1

Table S10. Antifungal activities of all compounds MIC (μg/mL)

Compounds	Fusarium oxysporum f. sp. cubense	*Fusarium spp.*	*Peronophythora litchii*	*Colletotrichum gloeosporioides*	*Hylocereus undatus*
**1**	>128	>128	>128	>128	>128
**2**	>128	>128	>128	>128	>128
**3**	>128	>128	>128	>128	>128
**4**	>128	>128	>128	>128	>128
**5**	>128	>128	>128	>128	>128
**6**	>128	>128	>128	>128	>128
**7**	>128	>128	>128	>128	>128
**8**	>128	>128	>128	>128	>128
**9**	>128	>128	>128	>128	>128
**10**	>128	>128	>128	>128	>128
**11**	>128	>128	>128	>128	>128
**12**	>128	>128	>128	>128	>128
**13**	>128	>128	>128	>128	>128
**14**	>128	>128	>128	>128	>128
**15**	>128	>128	>128	>128	>128
**16**	>128	>128	>128	>128	>128
**17**	>128	>128	>128	>128	>128
**18**	>128	>128	>128	>128	>128
**Carbendazim**	<1	<1	<1	<1	<1
